# Synthesis of Cis-Cisoid or Cis-Transoid Poly(Phenyl-Acetylene)s Having One or Two Carbamate Groups as Oxygen Permeation Membrane Materials

**DOI:** 10.3390/membranes10090199

**Published:** 2020-08-25

**Authors:** Yu Zang, Yinghui Lun, Masahiro Teraguchi, Takashi Kaneko, Hongge Jia, Fengjuan Miao, Xunhai Zhang, Toshiki Aoki

**Affiliations:** 1 Key laboratory of polymer matrix composites, Heilongjiang Province, College of Materials Science and Engineering, Qiqihar University, Wenhua Street 42, Qiqihar, Heilongjiang 161006, China; jiahongge11@hotmail.com (H.J.); zhangxunhai@163.com (X.Z.); toshaoki@eng.niigata-u.ac.jp (T.A.); 2 Department of Materials and Chemical Engineering, Hunan Institute of Technology, Hengyang, Hunan 421002, China; lunyinghui_888@163.com; 3 Chemistry and Chemical Engineering, Graduate School of Science and Technology, Niigata University, Ikarashi 2-8050, Nishi-ku, Niigata 950-2181, Japan; teraguti@eng.niigata-u.ac.jp (M.T.); kanetaka@gs.niigata-u.ac.jp (T.K.); 4 College of Communications and Electronics Engineering, Qiqihar University, Wenhua Street 42, Qiqihar, Heilongjiang 161006, China; miaofengjuan@qqhru.edu.cn

**Keywords:** polyphenylacetylene, cis-cisoid conformation, cis-transoid conformation, carbamate group, membrane-forming ability, solubility, oxygen permeation membrane

## Abstract

Three new phenylacetylene monomers having one or two carbamate groups were synthesized and polymerized by using (Rh(norbornadiene)Cl)_2_ as an initiator. The resulting polymers had very high average molecular weights (*Mw*) of 1.4–4.8 × 10^6^, with different solubility and membrane-forming abilities. The polymer having two carbamate groups and no hydroxy groups in the monomer unit showed the best solubility and membrane-forming ability among the three polymers. In addition, the oxygen permeability coefficient of the membrane was more than 135 times higher than that of a polymer having no carbamate groups and two hydroxy groups in the monomer unit with maintaining similar oxygen permselectivity. A better performance in membrane-forming ability and oxygen permeability may be caused by a more extended and flexible cis-transoid conformation and lower polarity. On the other hand, the other two new polymers having one carbamate group and two hydroxy groups in the monomer unit showed lower performances in membrane-forming abilities and oxygen permeabilities. It may be caused by a very tight cis-cisoid conformation, which was maintained by intramolecular hydrogen bonds.

## 1. Introduction

π-Conjugated polymers like polyacetylenes [1,2,3] have aroused interest because of their noteworthy physical properties, such as conductivity, organomagnetism, and optical nonlinear susceptibility. Among them, poly(substituted acetylene)s such as poly(substituted phenylacetylene)s are useful because of their stability in the air, possibility of a variety of derived structures, and good performances as separation membrane materials [4,5,6,7,8,9,10,11,12]. Poly(substituted phenylacetylene)s are generally rigid polymers and show good oxygen permselectivities. In addition, they are soluble and suitable as oxygen permeation membrane materials. Most of poly(substituted acetylene)s reported take cis-transoid conformation. On the other hand, the poly(substituted acetylene with two hydroxyl groups)s we reported take cis-cisoid conformation [13,14,15]. As a result, they are expected to have a more rigid backbone than cis-transoid poly(substituted acetylene)s. Therefore, cis-cisoid poly(substituted acetylene)s are very promising as better oxygen permeation membrane materials if their processability is good. In this paper, to discuss this factor—that is, the rigidity of the backbone—we compared the two acetylene polymers having these different conformations.

In general, polymers obtained by the polymerization of monosubstituted acetylenes using a (Rh(norbornadiene)Cl)_2_ (norbornadiene = nbd) catalytic system take a cis-transoidal loosely helical conformation [16,17,18,19]. We have been reporting poly(phenylacetylene)s taking a cis-cisoidal tightly helical conformation from monomers having two hydroxy groups, such as **4** (in Chart 1), using a similar catalytic system [13,14]. The cis-cisoidal tightly helical conformation of poly(**4**) was kept by an intramolecular hydrogen bond between the OH groups [14]. Although the monomer is very valuable, because it is the only monomer to give such polymers, the structures of suitable monomers giving such polymers are very limited. We also reported a phenylacetylene with two amido groups that can make hydrogen bonds instead of hydroxy groups as the second suitable monomer [15]. Therefore, it is important to find other suitable monomers having another functional groups that can make hydrogen bonds instead of hydroxy groups.

Since amino groups can make hydrogen bonds similarly to hydroxyl groups, monomers having amino groups are very promising. In addition, since amino groups are basic and important functional groups, therefore, polymers with amino groups are also important as reagents, catalysts, biocompatible or biodegradable materials, carbon dioxide permselective membranes, and so on [20,21,22,23,24,25,26,27]. However, the direct synthesis of amino group-containing poly(substituted acetylene)s by polymerizing the corresponding amino group-containing monomers using a rhodium complex as a catalyst has some problems. For example, the amino groups in monomers interact with the Rh catalyst so strongly [28,29] that the polymerization can be disrupted to yield only low *M_W_* polymers. Even if the polymerization proceeded, the resulting polymer should be insoluble due to the strong hydrogen bonds. Therefore, amino groups should be protected before rhodium complex-catalyzed polymerization. A carbamate group is a typical protecting group for amines, and it can weaken hydrogen bonds and makes the monomer more hydrophobic. In addition, since it has C=O and NH groups, it can still make hydrogen bonds. Therefore, carbamate group-containing poly(phenylacetylene)s can show similar characteristics and better solubility and membrane-forming properties than amino-containing poly(phenylacetylene)s. However, the solubility and the membrane-forming ability of polymers with cis-cisoidal tightly helical conformations such as poly(**4**) were not the best, although they were applied to oxygen permselective membranes [30]. It may be due to the tight cis-cisoidal helical main chain and rigid rod structures inducing some crystalline domains [30].

**Figure 1:**
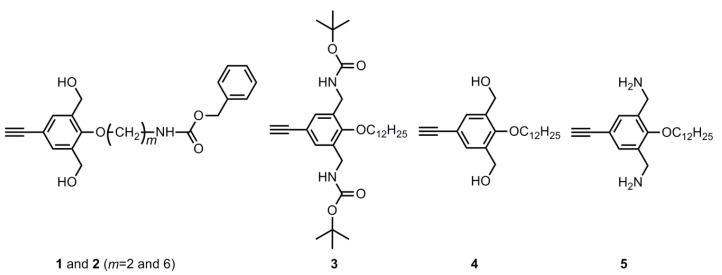
Chemical structures of new one or two carbamate-containing phenylacetylenes (**1**–**3**) and (**3** and **5**).

**Figure 2:**
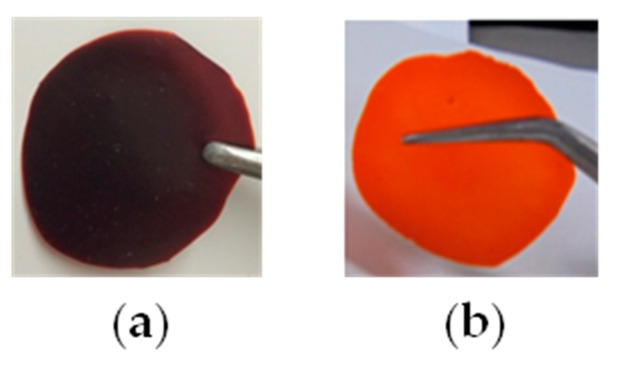
Photographs of membranes from (**a**) poly(**2**) and (**b**) poly(**3**).

**Figure 3:**
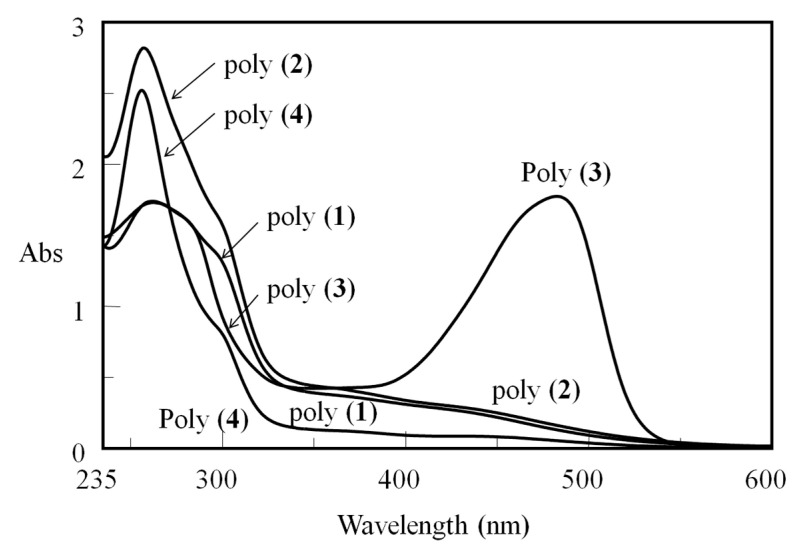
UV spectra of poly(**1**)–poly(**4**) in CHCl_3_ (c = 2.00mmoL/L).

In this study, in order to obtain carbamate group-containing poly(phenylacetylene)s having cis-cisoid or cis-transoid main chains that show high solubility and good membrane-forming abilities, we carried out the synthesis and polymerization of two kinds of novel phenylacetylene monomers—that is, two new monomers with one carbamate group and two hydroxy groups (Figure 1, **1**,**2**) and a new monomer containing two carbamate groups and no hydroxy groups (Figure 1, **3**). Then, we discuss some properties as oxygen permeation materials, such as the solubility, membrane-forming ability, and oxygen permeability of the resulting new polymers. The effects of the conformation of the polymer main chains on the properties are discussed.

## 2. Materials and Methods

### 2.1. Materials

All the solvents used for synthesis and polymerization of the monomers were distilled as usual. The polymerization initiator, (Rh(nbd)Cl)_2_ (nbd = 2,5 norbornadiene), purchased from Aldrich Chemical (Tokyo, Japan), was used as received.

### 2.2. Measurements

^1^H NMR (400 MHz) spectra were recorded on a JEOL LEOLEX-400 spectrometer (JEOL, Akishima, Japan). The average molecular weights (*M*_n_ and *M*_w_) were evaluated by gel permeation chromatography (GPC) by using JASCO liquid chromatography instruments (JASCO, Tokyo, Japan) with PU-2080, DG- 2080-53, CO-2060, UV-2070, and two polystyrene gel columns (Shodex KF-807 L, tetrahydrofuran (THF) eluent, polystyrene calibration, TCI, Tokyo, Japan). The infrared spectra (IR) were recorded on FT-IR-4200 (JASCO) (JASCO, Tokyo, Japan). UV-vis spectra were measured with a JASCO V-550 spectropolarimeter (JASCO, Tokyo, Japan).

### 2.3. Synthesis of Monomer ***1***

#### 2.3.1. N-Benzyloxycarbonyl-2-aminoethanol (**10**, m = 2) 

Benzyl chloroformate (6.40 mL, 45.4 mmoL) in diethyl ether (14.0 mL) was added dropwise to a solution of 2-aminoethanol (2.70 mL, 45.4 mmoL) in 10% aqueous Na_2_CO_3_ (54.0 mL) at 0 °C and stirred for 1.5 h. The reaction mixture was acidified with 10% HCl at 0 °C to give precipitates that were filtered and washed with H_2_O to give **10** as a white crystal. [31] Yield: 35.2% (3.06 g). ^1^H NMR (400 MHz, CDCl_3_, TMS, δ): 7.32 (m, 5H, Ph*H*), 5.15 (br, 1H, CH_2_N*H*CO), 5.09 (s, 2H, PhC*H*_2_OCO), 3.70 (q, 2H, OHC*H*_2_CH_2_), 3.35 (q, 2H, CH_2_C*H*_2_NH), 2.17 (br, 1H, CH_2_O*H*).

#### 2.3.2. N-Benzyloxycarbonyl-2-bromoethylamine (**11**, m = 2)

A flask was charged with **10** (9.76 g, 50.0 mmoL), methanesulfonyl chloride (4.65 mL, 60.0 mmoL,), and CH_2_Cl_2_ (150 mL). To this stirring solution, Et_3_N (9.01 mL, 65.0 mmoL) was added. Stirring was continued for 45 min, and then, LiBr (43.5 g, 500 mmoL) and acetone (150 mL) were added. The reaction mixture was stirred for an additional 21.5 h, and then, the solvents were removed by rotary evaporation. The contents were partitioned between Et_2_O (100 mL) and H_2_O (65.0 mL), and the Et_2_O layer was washed with brine, dried over anhydrous Na_2_SO_4_, filtered, evaporated to dryness, and got the brown liquid product. Yield: 90.9% (2.98 g). [32] ^1^H NMR (400 MHz, CDCl_3_, TMS, δ): 7.35 (m, 5H, Ph*H*), 5.18 (br, 1H, CH_2_N*H*CO), 5.10 (s, 2H, PhC*H*_2_OCO), 3.60 (q, 2H, CH_2_C*H*_2_NH), 3.45 (t, 2H, BrC*H*_2_CH_2_).

#### 2.3.3. 4-(N-Benzyloxycarbonyl-2-ethylamino)benzyloxy-3,5-bis(hydroxymethyl)phenylacetylene (**1**, m = 2)

The solution of **11** (0.736 g, 2.87 mmoL), **9** (0.500 g, 2.81 mmoL), and potassium carbonate (0.620 g, 4.49 mmoL) in N,N-dimethyformamide (DMF) (15.0 mL) was refluxed for 48 h and cooled to room temperature. Then, the mixture was filtered, and the solvent in the filtrate was removed by evaporation. The crude product was purified by silica-gel column chromatography to give 1 as a white solid. Yield: 23.1% (0.231 g). Retention volumes (R_f_) = 0.20 (ethyl acetate/hexane = 1/1). ^1^H NMR (400 MHz, CDCl_3_, TMS, δ): 7.45 (s, 2H, Ph*H*), 7.32 (m, 5H, Ph*H*), 5.60 (br, 1H, CH_2_N*H*CO), 5.10 (s, 2H, PhC*H*_2_OCO), 4.62 (d, 4H, Ph(C*H*_2_OH)_2_), 4.01 (t, 3H, PhOC*H*_2_CH_2_), 3.58 (q, 2H, CH_2_C*H*_2_NH), 3.02 (s, 1H, *H*C≡C), 2.03 (t, 2H, Ph(CH_2_O*H*)_2_). IR (cm^−1^, KBr): 3380 (OH), 3311 (NH), 3298 (H–C≡), 1692 (C=O), 1267 (C–O), 1051 (C–N). (For the synthesis of **9**, see S1.1–S1.4 in the Supporting Information.) 

### 2.4. Synthesis of Monomer ***2***

#### 2.4.1. N-(Benzyloxycarbonyl)-6-amino-1-hexanol (**10**, m = 6)

The synthesis procedure for N-(benzyloxycarbonyl)-6-amino-1-hexanol (**10**, *m* = 6) was similar to N-benzyloxycarbonyl-2-aminoethanol (**10**, *m* = 2) to give a white crystal. Yield: 44.8% (4.82 g). ^1^H NMR (400 MHz, CDCl_3_, TMS, δ): 7.35 (m, 5H, Ph*H*), 5.08 (s, 2H, PhC*H*_2_OCO), 4.72 (br, 1H, CH_2_N*H*CO), 3.61 (q, 2H, HOC*H*_2_CH_2_), 3.19 (q, 2H, CH_2_C*H*_2_NH), 1.55–1.31 (m, 8H, HOCH_2_(C*H*_2_)_4_CH_2_).

#### 2.4.2. N-(Benzyloxycarbonyl)-6-bromohexylamine (**11**, m = 6)

The synthesis procedure for N-(benzyloxycarbonyl)-6-bromohexylamine (**11**, *m* = 6) was similar to N-benzyloxycarbonyl-2-bromoethylamine (**11**, *m* = 2) to give a brown crystal. Yield: 73.7% (2.30 g). ^1^H NMR (400 MHz, CDCl_3_, TMS, δ): 7.35 (m, 5H, Ph*H*), 5.08 (s, 2H, PhC*H*_2_OCO), 4.72 (br, 1H, CH_2_N*HCO*), 3.40 (t, 2H, BrC*H*_2_CH_2_), 3.17 (q, 2H, CH_2_C*H*_2_NH), 1.83(m, 2H, BrCH_2_C*H*_2_CH_2_), 1.55–1.31 (m, 6H, BrCH_2_CH_2_(C*H*_2_)_3_CH_2_).

#### 2.4.3. 4-(N-Benzyloxycarbonyl-6-hexylamino)benzyloxy-3,5-bis(hydroxymethyl)phenylacetylene (**2**, m = 6)

The synthesis procedure for monomer **2** was similar to monomer **1** to give a white solid. Yield: 32.2% (0.370 g). R_f_ = 0.30 (ethyl acetate/hexane = 1/1). ^1^H NMR (400 MHz, CDCl_3_, TMS, δ): 7.45 (s, 2H, Ph*H*), 7.32 (m, 5H, Ph*H*), 5.07 (s, 2H, PhC*H*_2_OCO), 4.81 (br, 1H, CH_2_N*HCO*), 4.67(d, 4H, Ph(C*H*_2_OH)_2_), 3.85 (t, 2H, PhOC*H*_2_CH_2_), 3.20 (q, 2H, CH_2_C*H*_2_NH), 3.02 (s, 1H, *H*C≡C), 2.16 (t, 2H, Ph(CH_2_O*H*)_2_), 1.77 (m, 2H, PhOCH_2_C*H*_2_CH_2_), 1.52-1.36 (m, 6H, CH_2_(C*H*_2_)_3_CH_2_NH). IR (cm^−1^, KBr): 3380 (OH), 3356 (NH), 3304 (H–C≡), 1681 (C=O), 1268 (C–O), 1060 (C–N).

### 2.5. Synthesis of Monomer ***3***

#### 2.5.1. 4-Dodecyloxy-3,5-bis(hydroxymethyl)phenylacetylene (**4**)

According to the literature [23], **4** was synthesized to give a white solid. Yield: 69.6% (1.25g). R_f_ = 0.24 (ethyl acetate/hexane = 1/4). ^1^H-NMR(400MHZ, CDCl_3_, TMS, δ): 7.46 (s, 2H, Ph*H*), 4.68 (d, 4H, Ph(C*H*_2_OH)_2_), 3.88 (t, 2H, OC*H*_2_CH_2_(CH_2_)_9_CH_3_), 3.00 (s, 1H, *H*C≡C), 1.96 (t, 2H, (CH_2_O*H*)_2_), 1.79 (dm, 2H, OCH_2_C*H*_2_CH_2_), 1.50-1.20 (m, 18H, OCH_2_CH_2_(C*H*_2_)_9_CH_3_), 0.883 (t, 3H, OCH_2_CH_2_(CH_2_)_9_C*H*_3_). IR (KBr): 3300 (OH), 3250 (H–C≡), 2840 (CH), 2095 (C≡C).

#### 2.5.2. 4-Dodecyloxy-3,5-bis(bromomethyl)phenylacetylene (**12**)

The solution of **4** (1.00 g, 2.89 mmoL), carbon tetrabromide (3.26 g, 9.82 mmoL), and triphenylphosphine (2.27 g, 8.67 mmoL) in dichloromethane (40.0 mL) was stirred at 0 °C for 4 h. Saturated solution of NaHCO_3_ was added to the mixture, and then, liquid–liquid separation was carried out by tap funnel. The organic layer was dried over anhydrous MgSO_4_, evaporated, and the residue was purified by column chromatography to give a white solid. Yield: 77.0% (1.05 g). R_f_ = 0.17 (hexane). ^1^H NMR (400 MHz, CDCl_3_, TMS, δ): 7.50 (s, 2H, Ph*H*), 4.49 (s, 4H, Ph(C*H*_2_Br)_2_), 4.09 (t, 2H, OC*H*_2_CH_2_(CH_2_)_9_CH_3_), 3.06 (s, 1H, *H*C≡C), 1.89 (m, 2H, OCH_2_C*H*_2_(CH_2_)_9_CH_3_), 1.27–1.55 (m, 18H, OCH_2_CH_2_(C*H*_2_)_9_CH_3_), 0.88 (t, 3H, OCH_2_CH_2_(CH_2_)_9_C*H*_3_).

#### 2.5.3. 4-Dodecyloxy-3,5-bis(azidomethyl)phenylacetylene (**13**)

A solution of **12** (0.500 g, 1.06 mmoL) and NaN_3_ (275 mg, 4.24 mmoL) in DMF (4.50 mL) was stirred at room temperature. After stirring of the mixture for 48 h at room temperature, the mixture was poured into water and extracted with dichloromethane. The organic layer was washed with water, brine, and dried over anhydrous MgSO_4_. The organic solvent was removed by evaporation, and the crude product was purified by silica-gel column chromatography to give a pale-yellow oil. Yield: 95.0% (403 mg) [33]. R_f_ = 0.4 (ethyl/hexane = 1/20). ^1^H NMR (400 MHz, CDCl_3_, TMS, δ): 7.50 (s, 2H, Ph*H*), 4.49 (s, 4H, Ph(C*H*_2_N_3_)_2_), 4.09 (t, 2H, OC*H*_2_CH_2_(CH_2_)_9_CH_3_), 3.06 (s, 1H, *H*C≡C), 1.89 (m, 2H, OCH_2_C*H*_2_(CH_2_)_9_CH_3_), 1.27–1.55 (m, 18H, OCH_2_CH_2_(C*H*_2_)_9_CH_3_), 0.88 (t, 3H, OCH_2_CH_2_(CH_2_)_9_C*H*_3_).

#### 2.5.4. 4-Dodecyloxy-3,5-bis(aminomethyl)phenylacetylene (**5**) 

To a solution of **13** (400 mg, 1.01 mmoL) in THF (15.0 mL), a mixture of LiAlH_4_ (95.8 mg, 2.53 mmoL) and THF (10.0 mL) was added dropwise at 0° C. The mixture was stirred for 24 h at room temperature. When the reaction finished, H_2_O was added dropwise at 0° C. A saturated aqueous solution of NaOH (10.0 mL) was added dropwise to the mixture and stirred for 30 min. After the mixture was filtered, the organic solvent was removed by evaporation. The crude product was washed with a saturated solution of NaCl. The organic layer was dried over anhydrous MgSO_4_ and then evaporated to give a yellow liquid. Yield: 91.5% (0.541 g) [33]. ^1^H NMR (400 MHz, CDCl_3_, TMS, δ): 7.36 (s, 2H, Ph*H*), 3.81 (s, 4H, Ph(C*H*_2_NH_2_)_2_)_,_ 3.80(t, 2H, PhOC*H*_2_CH_2_), 3.00 (s, 1H, *H*C≡C), 1.81 (m, 2H, PhOCH_2_C*H*_2_CH_2_), 1.57 (br, 4H, Ph(CH_2_N*H*_2_)_2_), 1.27–1.55 (m, 18H, OCH_2_CH_2_(C*H*_2_)_9_CH_3_), 0.88 (t, 3H, OCH_2_CH_2_(CH_2_)_9_C*H*_3_).

#### 2.5.5. 4-Dodecyloxy-3,5-bis(tert-butoxycarbonylamino)) Phenyl Acetylene (**3**)

A THF (12 mL) solution of **5** (20 mg, 0.058 mmoL), di-tert-butyl dicarbonate ((Boc)_2_O) (27.9 mg, 0.128 mmoL), and 4-dimethylaminopyridine (DMAP) (0.7 mg) was refluxed for 6 h and then cooled to room temperature. THF was removed by evaporation, and the crude product was purified by silica-gel column chromatography to give monomer **3** as a white solid. Yield: 25.3% (7.10 mg). R_f_ = 0.45 (hexane/ethyl acetylene = 4/1). ^1^H NMR (400 MHz, CDCl_3_, TMS, δ): 7.34 (s, 2H, Ph*H*), 4.84 (br, 2H, Ph(CH_2_N*H*CO)_2_), 4.32 (d, 4H, Ph(C*H*_2_NH)_2_), 3.77 (t, 2H, PhOC*H*_2_CH_2_), 3.01 (s, 1H, *H*C≡C), 1.80 (m, 2H, OCH_2_C*H*_2_CH_2_), 1.44 (s, 18H, ((OC*H*_3_)_3_)_2_), 1.27-1.55 (m, 18H, CH_2_(C*H*_2_)_9_CH_3_), 0.88 (t, 3H, CH_2_C*H*_3_). IR (cm^−1^, KBr): 3370 (NH), 3298 (≡C–H), 1691 (C=O), 1480 (CH), 1168 (C–O), 1096 (C–N).

### 2.6. Polymerization of Monomers ***1***–***3***

**Scheme 1:**
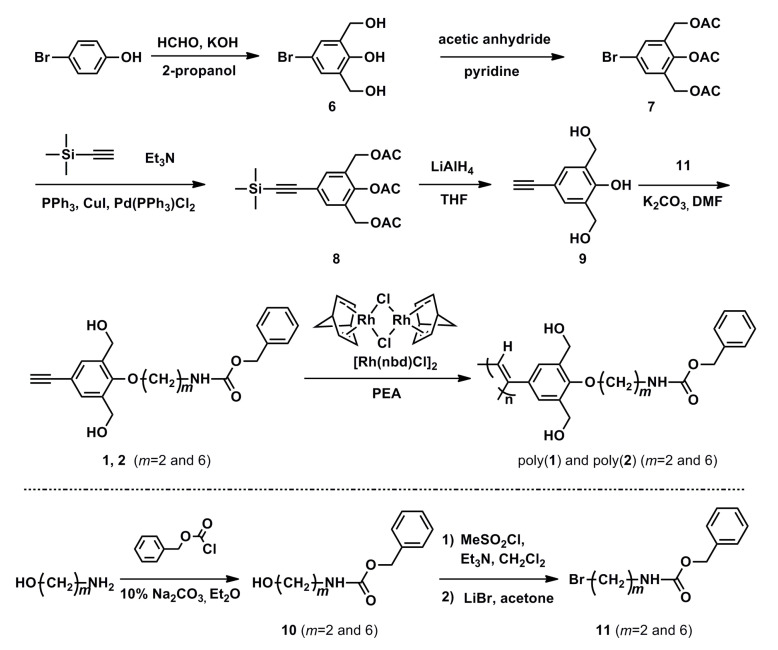
Synthetic route to poly(**1**) and poly (**2**).

**Scheme 2:**
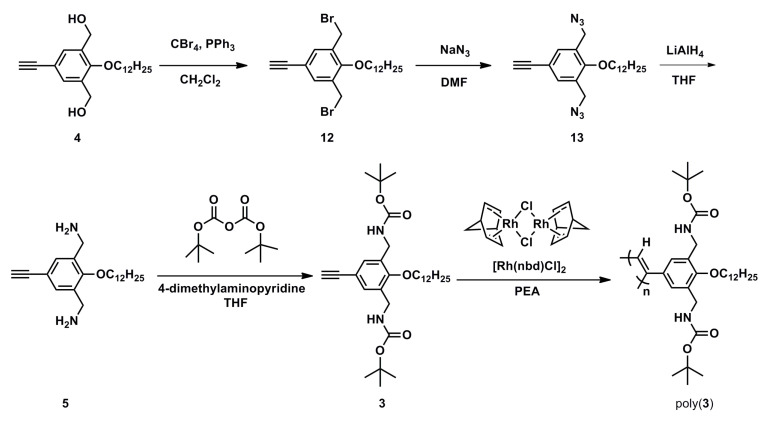
Synthetic route to poly(**3**).

A typical procedure for **1** was as follows (Scheme 1 and Scheme 2): A dry THF (0.350 mL) solution of (Rh(nbd)Cl)_2_ (0.322 mg, 0.700 µmol) and 1-phenethylamine (PEA) (17.9 µL, 0.141 mmoL) was added to a dry THF (0.350 mL) solution of 1 (25.0 mg, 0.070 mmoL). The reaction solution was stirred at room temperature for 8 h. The crude polymer was purified by reprecipitation of the THF solution into a large amount of methanol, and the formed solid was dried in vacuo to give poly(**1**).

Other polymerizations of monomers **2** and **3** were carried out similarly. (Figures S1–S20)

Poly(**1**) ^1^ H NMR(400 MHz, dimethylsulfoxide-d_6_ (DMSO-d_6_), δ): 7.37 (br, Ph*H*), 6.68 (br, *cis* proton in the main chain), 5.70 (br, CH_2_N*H*CO), 5.07–4.63 (br, PhC*H*_2_OCO, Ph(C*H*_2_OH)_2_), 4.28 (br, Ph(CH_2_O*H*)_2_). IR (cm^−1^, KBr): 3334 (NH, OH), 1690 (C=O), 1480 (CH), 1261 (C–O), 1096 (C–N).
Poly(**2**) ^1^ H NMR(400 MHz, DMSO-d_6_, δ): 7.38–7.27 (br, Ph*H*), 6.73 (br, *cis* proton in the main chain), 5.76 (br, CH_2_N*H*CO), 5.29–4.68 (br, PhC*H*_2_OCO, Ph(C*H*_2_OH)_2_), 4.33 (br, Ph(CH_2_O*H*)_2_), 3.04 (br, CH_2_C*H*_2_NH_,_ PhOC*H*_2_CH_2,_), 1.46–1.30 (t, 6H, CH_2_(C*H*_2_)_3_NH). IR (cm^−1^, KBr): 3334 (NH, OH), 1690 (C=O), 1480 (CH), 1261 (C–O), 1096 (C–N).
Poly(**3**) ^1^ H NMR(400 MHz, CDCl_3_, TMS, δ): 7.50 (br, Ph*H*), 5.10 (br, Ph(CH_2_N*H*CO)_2_), 3.48 (br, Ph(C*H*_2_NH)_2_, PhOC*H*_2_CH_2_), 2.02 (br, PhOCH_2_C*H*_2_CH_2_), 1.66–1.54 (br, ((OC*H*_3_)_3_)_2_), 1.23–1.09 (br, CH_2_(C*H*_2_)_9_CH_3_), 0.86 (br, CH_2_C*H*_3_). IR (cm^−1^, KBr): 3370 (N–H), 1691 (C=O), 1480 (CH), 1168 (C–O), 1096 (C–N).

### 2.7. Membrane Preparation

A typical membrane fabrication method for poly(**1**) was as follows: A solution of the poly(1) (0.060-10.0 wt%) in DMF (40.0 mg/mL) was cast on a poly(tetrafluoroethylene) sheet (4 cm^2^). After evaporating of the solvent for 12 h at 25 °C, the membranes were detached from the sheet and dried in a vacuum oven for 24 h at 60 °C. Thickness (L) of the membranes was 50.0–80.0 μm ± 0.5 μm. Other polymer membranes were prepared similarly.

### 2.8. Estimation of Polymers as Oxygen Permeation Membranes

#### 2.8.1. Membrane Strength

Maximum flexural stresses (σ/kPa) of membranes were calculated according to the following equation:

(1) $$\sigma =\frac{3FL}{2bd^{2}}$$

where F, *L*, *b*, and *d* are the load, length of the support span, width of membrane, and thickness of membrane, respectively (Figure S21).

#### 2.8.2. Oxygen Permeation

Oxygen and nitrogen permeability coefficients (*P*_O2_ and *P*_N2_: cm^3^(STP) · cm · cm^−2^ · s^−1^ · cmHg^−1^) (STP = standard temperature and pressure) and the oxygen separation factor (*α* = *P*_O2_/*P*_N2_) were measured by a gas chromatographic method by using YANACO GTR-10, according to reference [34]. The *P*_O2_ and *P*_N2_ were calculated by the following equation:

(2) $$P=\frac{Q\times L}{A\times \Delta P\times t}$$

where Q, *L*, A, ΔP, and t are the amount of the permeated gas, the thickness of the membrane, the permeation area of the membrane, the pressure difference across the membrane, and the permeation time, respectively. Disc-type membranes were used. The A and *L* of the membranes were 3.14 cm^2^ and around 60-80 μm, respectively. The ΔP was 1 atm, and the measurement temperature was 25 °C.

The diffusion coefficient (D: cm^2^·σ^−1^) was calculated by the time-lag method represented by *D* = *L* ^2^/6*θ*, where *L* (cm) is the thickness of the membrane, and *θ*(s) is the time-lag.

## 3. Results and Discussion

### 3.1. Synthesis of Monomers ***1***–***3***

To obtain polymers having one or two carbarmate groups, two different types of new phenylacetylene monomers were synthesized according to Scheme 1 and Scheme 2. The first type of the monomers contains two hydroxy groups and one carbarmate group (Figure 1, **1**,**2**). By five-step reactions from 4-bromophenol, monomers **1** and **2** were synthesized successfully (Scheme 1). The two novel monomers were purified by silica-gel column chromatography, and the total yields of monomers **1** and **2** were 5.5% and 7.7%, respectively. The second type of the novel monomers contains two carbarmate groups (Figure 1, **3**). It was synthesized by a four-step reaction from **4** according to our previous report [13] (Scheme 2). Monomer **3** was purified by silica-gel column chromatography, and the total yield was 7.6%. In addition, monomer **5** was synthesized from **4** by a three-step reaction and was purified by vacuum-drying as a yellow liquid in a total yield of 69.2% (Scheme 2). The polarity of the monomers showed a significant impact on the polymerization results, such as yields, solubility, membrane-forming ability, and so on (Table 1 and Table 2). The decreasing order of polarity of the five monomers was **5** > **1** > **2** > **4** > **3**, judging from the retention volumes (R_f_) of the monomers on the silica-gel thin-layer chromatography (TLC) using ethyl acetate/hexane (1/1) as an eluent (Table 1). When the two hydroxy (in monomer **4**) or amino groups (in monomer **5**) were replaced with two carbamate groups, the polarity of the monomer (monomer **3**) decreased largely.

### 3.2. Synthesis of poly(***1***)–poly(***3***)

Monomers 1–3 were polymerized by using (Rh(nbd)Cl)_2_/1-phenethylamine(PEA) (nbd = norbornadiene) as a catalytic system to give poly(**1**)–poly(**3**) (Scheme 1 and Scheme 2). The polymerization results are shown in Table 1. The yields of the resulting polymers were 38.0–72.4%, and they had very high molecular weights of 1.4–4.8 × 10^6^. Polymerization yields of **1** and **2**, which contain two hydroxy groups and one carbarmate group, were lower (Table 1, 1,2, 52.4% and 38.0%) than that of **3,** which has two carbarmate groups instead of two hydroxy groups (Table 1, 3, 72.4%). This result may be because the high polarity of **1** and **2** decreased the efficiency of the rhodium catalytic system during the polymerization. In addition, the solubility of the monomers and polymers of **1** and **2** were lower than that of **3**. It may be another reason for the low yields of poly(**1**) and poly(**2**). The yield of poly(**4**) was lower similar than those for poly(**1**) and poly(**2**) due to the two hydroxy groups (Table 1, 4, 43.2%). For poly(**5**), the yield was quite low; only 4.6% of the monomer was converted to the polymer (Table 1, 5). Since monomer **5** has two amino groups, it may be interacting with the rhodium catalyst to prevent the polymerization. Due to the high polarity, the resulting poly(**5**) was insoluble in the common solvent.

### 3.3. Effects of the Main Chain Conformation on the Solubility and Membrane Strengths

The three new synthetic polymers (poly(**1**)–poly(**3**)) showed different solubilities, as shown in Table 2. Poly(**1**) and poly(**2**) have two hydroxy groups and one carbamate group in the monomer unit and different lengths of methylene spacers (m) between the carbamate and phenoxy group. They showed low solubility in THF and DMF and insolubility in toluene. On the other hand, poly(**3**), which has two carbamate groups instead of the two hydroxy groups in poly(**1**) and poly(**2**), showed good solubility not only in THF but, also, in toluene (Table 2, 3). Judging from the solubilities for the polymers and R_f_ values for the monomers in the TLC analysis (Table 1), 3 and poly(**3**) were more hydrophobic than **1** and poly(**1**) and **2** and poly(**2**).

Poly(**1**)–poly(**3**) showed different membrane-forming abilities, as shown in Table 2. Although self-standing membranes could be fabricated from the DMF solution of poly(**1**) and poly(**2**), they were brittle and weak. The maximum flexural stresses for poly(**1**) and poly(**2**) were 0.968 and 2.40 × 10^3^ KPa (Table 2, 1,2), respectively. Since poly(**2**) showed a little better membrane-forming ability than poly(**1**), the longer spacer (m = 6) in poly(**2**) enhanced the flexibility. On the other hand, the membrane strength of poly(**3**) was much higher (the maximum flexural stress was 53.6 × 10^3^ KPa) and 55 times higher than that of poly(**1**) (Table 2, 3).

Since poly(**1**) and poly(**2**) membranes having carbamate groups showed the same red color as poly(**4**) having no carbamate groups (Table 2 and Figure 2), the main chains of the two new polymers having two hydroxy groups in the monomer unit had a very tight cis-cisoid conformation similar to poly(**4**) [16,17,18,19]. We previously reported that this conformation tended to decrease the solubility of the polymers and flexibility of the polymer membranes. On the other hand, poly(**3**) having no hydroxy groups and carbamate groups was orange (Table 2). It was suggested that the polymer had a more extended and flexible cis-transoid conformation, which tended to increase the solubility of the polymers and flexibility of the polymer membranes. In order to confirm the main chain conformations of the three new polymers having one or two carbamate groups (poly(**1**), poly(**2**), and poly(**3**)), UV-vis spectra were measured for them, together with poly(**4**) having no carbamate groups (Figure 3). Since poly(**1**) and poly(**2**) showed a similar UV-vis pattern to poly(**4**), whose main chain had a very tight cis-cisoid conformation maintained by intramolecular hydrogen bonds reported by our group [13,14], we concluded that poly(**1**) and poly(**2**) took very tight cis-cisoid conformations. On the other hand, the UV-vis spectrum of poly(**3**) showed different absorption bands from those of poly(**1**) and poly(**2**). The peak around 480nm indicates that the main chain of poly(**3**) consists of a more extended and flexible cis-transoid conformation (Appendix A, 1st item).

**Figure 4:**
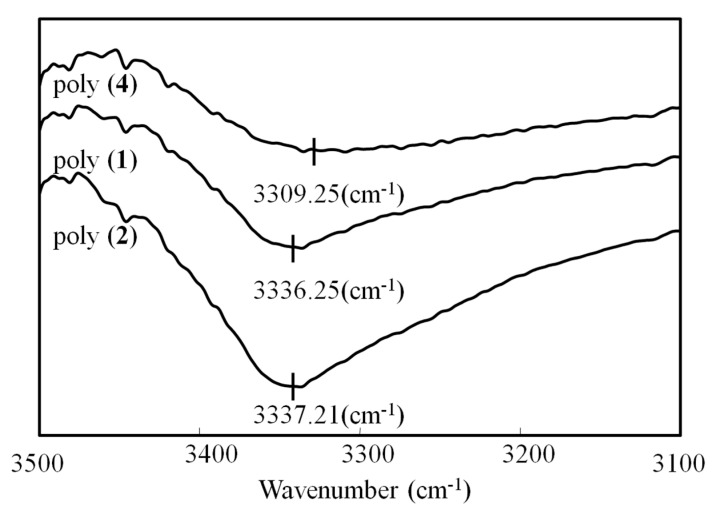
Solution IR spectra of poly(**1**), poly(**2**), and poly(**4**) in CHCl_3_ (2.00 mmoL/L) at room temperature.

**Figure 5:**
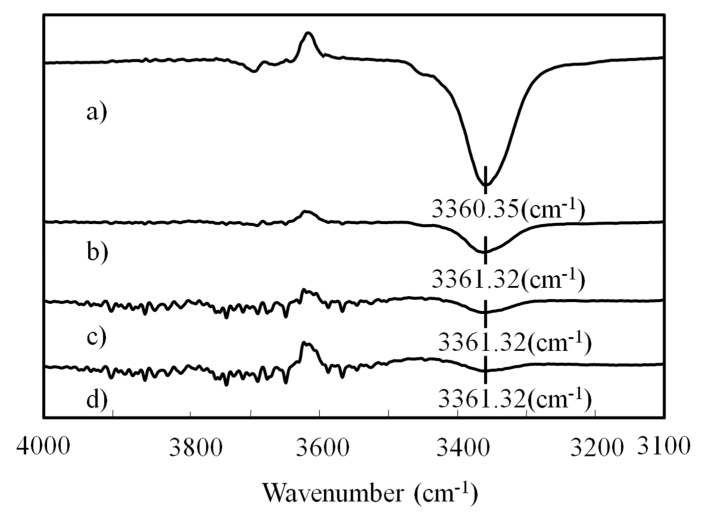
Solution IR spectra of poly(**3**) in CHCl_3_ at room temperature: (**a**) 8.0 mmoL/L, (**b**) 2.0 mmoL/L, (**c**) 1.0 mmoL/L, and (**d**) 0.50 mmoL/L.

In order to discuss hydrogen bonds that can support the very tight cis-cisoid conformations in the new polymers, the IR spectra of poly(**1**) and poly(**2**), together with poly(**4**), which has two hydroxy groups in its monomer unit, were measured in CHCl_3_ (2.00 mmoL/L) (Figure 4). The stretching vibration bands of O–H were observed around 3336 and 3337cm^−1^ for poly(**1**) and poly(**2**), respectively. Since they were similar to the stretching vibration band of O–H for poly(**4**) having no carbamate groups, it is suggested that poly(**1**) and poly(**2**) also have similar hydrogen bonds to poly(**4**). Therefore, they could have cis-cis conformation. In order to discuss hydrogen bonds between the carbamate groups in poly(**3**), the IR spectra in CHCl_3_ were measured in different concentrations (0.50–8.0 mmoL/L) (Figure 5). No intramolecular hydrogen bonds were found, because the stretching vibration band of N–H around 3361 cm^−1^ has almost no shift by changing the concentration. Therefore, poly(**3**) could not take a very tight cis-cisoid conformation supported by hydrogen bonds between the carbamate groups, and instead, took a more extended and flexible cis-transoid conformation.

In conclusion, poly(**3**) had the best solubility and the best membrane-forming ability among the three new polymers, because it had a flexible cis-transoid conformation without hydrogen bonds, while poly(**1**) and poly(**2**) had less solubility and less membrane-forming abilities than poly(**3**), because they had rigid cis-cisoid conformations maintained by hydrogen bonds (Appendix A, 2nd item). In addition, the polarity of the polymers also affected the solubility. Poly(**1**) and poly(**2**) having two hydroxy groups showed higher polarity than poly(**3**). Therefore, the solubility and the membrane-forming ability of poly(**3**) was the best.

### 3.4. Oxygen Permeability of the Membranes from the New Polymers 

The oxygen and nitrogen permeability coefficients (*P*_O2_ and *P*_N2_) through the membranes of polyphenylacetylenes (poly(**2**) and poly(**3**)) having one or two carbamate groups were newly determined, as shown in Table 3 and Figure S22. (Poly(**1**) was too weak to resist one atom pressure difference during the oxygen permeation measurement.) The polymer membranes of poly(**2**) and poly(**3**) showed much higher permeability coefficients (*P*_O2_ = 188 and 420, respectively) than that of poly(**4**) (*P*_O2_ = 3.09) having no carbamate group. Poly(**3**) showed about 135 times higher *P*_O2_ than poly(**4**) and about 2.2 times higher *P*_O2_ than poly(**2**). In other words, by introducing one relatively hydrophobic carbamate group to poly(**4**), poly(**2**) had a much higher *P*_O2_ than poly(**4**), and by replacing two hydroxy groups in poly(**4**) with two relatively bulky carbamate groups, poly(**3**) had a much higher *P*_O2_ than poly(**4**). In addition, poly(**2**) and poly(**3**) having higher *P*_O2_ than poly(**4**) showed only a small drop in oxygen permselectivity (*P*_O2_/*P*_N2_) compared with poly(**4**) (See Table 3 and Figure S22).

To discuss the reason for this change in *P*_O2_ and *P*_O2_/*P*_N2_, the oxygen diffusion coefficient (*D*_O2_) and *D*_O2_/*D*_N2_ values were determined from the time lags (Table 3). The higher *P*_O2_ values of the poly(**2**) and poly(**3**) membranes were caused by the higher *D*_O2_ values. Since the *P*_O2_/*P*_N2_ values mainly depend not on *D*_O2_/*D*_N2_ values, which were almost a unit (=1.0), but *S*_O2_/*S*_N2_ values, which were almost the same (=2.5), the *P*_O2_/*P*_N2_ values (=*D*_O2_/*D*_N2_ x *S*_O2_/*S*_N2_) did not change largely. In other words, the permeability depends on the diffusivity, and the selectivity depends on the solution selectivity (Figure S23).

In conclusion, poly(**3**) taking a cis-transoid conformation gave a higher *P*_O2_ because of a higher *D*_O2_ than poly(**2**) and poly(**4**) taking cis-cisoid conformations. The three polymers showed similar *P*_O2_/*P*_N2_ values, because *P*_O2_/*P*_N2_ mainly depend on *S*_O2_/*S*_N2_, which were almost similar among the three polymers.

## 4. Conclusions

Three new phenylacetylene monomers (**1**–**3**) having one or two carbamate groups were successfully synthesized, and they gave polymers in 38.0–72.4% yields by polymerization using (Rh(norbornadiene)Cl)_2_ as a catalyst (Appendix A, 3rd item). The polymers had very high average molecular weights (*Mw*) of 1.4–4.8 × 10^6^, with different solubility and membrane-forming abilities. Poly(**3**) having two carbamate groups and no hydroxy groups in the monomer unit showed the best solubility and membrane-forming ability among the three new polymers. In addition, the oxygen permeability coefficient (*P*_O2_) of the membrane of poly(**3**) was 420 barrer, which was more than 135 times higher than that of poly(**4**) having no carbamate group and hydroxy groups with maintaining a similar oxygen permselectivity (*P*_O2_/*P*_N2_). The better performance in membrane-forming ability and oxygen permeability for poly(**3**) may be caused by the more extended and flexible cis-transoid conformations and lower polarity (Figures S24 and S25). In other words, a higher solubility gave good dense membranes without defects. On the other hand, the other two polymers having one carbamate group and two hydroxy groups in the monomer unit (poly(**1**) and poly(**2**)) showed less performance in membrane-forming abilities and oxygen permeabilities. It may be caused by a very tight cis-cisoid conformation that was maintained by intramolecular hydrogen bonds and a higher polarity.

## Figures and Tables

**Table 1:** The polymerization results of monomers **1**–**5** ^a.^

No.	Monomer ^b^	R_f_^c^	Solvent	Yield (%) ^d^	*M*_w_ (*×*10^6^) ^e^	*M*_w_/*M*_n_^e^
1	**1**	0.20	THF	52.4	4.80	2.32
2	**2**	0.30	THF	38.0	1.40	6.50
3	**3**	0.93	toluene	72.4	2.90	4.31
4	**4**	0.80	toluene	43.2	3.10	5.40
5	**5**	0.00	toluene	4.60	- ^f^	- ^f^

^a^ At room temperature for 8 h, (Monomer) = 0.100 mol/L, (Monomer)/((Rh[nbd]Cl)_2_) = 100, and ((Rh[nbd]Cl)_2_)/(1-phenethylamine) = 1/200. ^b^ For the codes, see Chart 1. ^c^ Retention volumes of the monomers on silica-gel thin-layer chromatography (TLC) using eltylacetate/hexane = 1/1 as an eluent. ^d^ Methanol insoluble part. ^e^ Determined by the gel permeation chromatography (GPC) correlating polystyrene standard with a THF eluent. ^f^ No data due to insolubility of the polymer (Table 2).

**Table 2:** Solubility of poly(**1**)–poly(**5**) and the characterization of their membranes.

No.	Polymer	Solubility ^a^			Membrane-Forming Ability ^b^	Maximum Flexural Stress (*×*10^3^) (σ/KPa) ^c^	Color ^d^
		Toluene	THF	DMF			
1	poly(**1**)	−	+	+	+ ^e^	0.968	deep red
2	poly(**2**)	−	+	+	++ ^e^	2.40	deep red
3	poly(**3**)	++	++	−	+++ ^f^	53.6	orange
4	poly(**4**)	+	+	−	+ ^f^	4.29	deep red
5	poly(**5**)	−	−	−	− ^g^	− ^g^	yellow ^h^

_a_ ++: Highly soluble, +: soluble, and -: insoluble. _b_ +++: Tough, ++: flexible, and +: brittle. _c_ See Figure S21. _d_ In a membrane state (see Figure 2). _e_ The membranes were fabricated by solvent cast using a DMF solution. _f_ The membranes were fabricated by solvent cast using a toluene solution. _g_ No data due to the insolubility. _h_ In a powder state.

**Table 3:** Oxygen permeation behavior of the membranes of poly(**2**)–poly(**4**) ^a.^

No.	Membrane ^a^	*P*_O2_ (Barrer) ^b^	*P*_O2_/*P*_N2_	*D*_O2_^c^	*D*_O2_/*D*_N2_	*S*_O2_^d^	*S*_O2_/*S*_N2_
1	poly(**2**)	188	2.56	11.6	1.05	16.1	2.44
2	poly(**3**)	420	2.70	184	1.06	2.28	2.54
3	poly(**4**)	3.09	3.04	3.41	1.25	0.909	2.44

^a^ For the codes, see Chart 1. ^b^ 1 barrer = 10^−10^ cm^3^(STP) cm cm^−2^ s^−1^ cmHg^−1^. ^c^ In × 10^−8^ cm^2^/s. ^d^ In × 10^−^^2^ cm^3^(STP) cm^3^ cmHg^−^^1^.

## Supplementary Materials

The following are available online at https://www.mdpi.com/2077-0375/10/9/199/s1: Synthesis of compounds 6–9. Scheme S1: Polymerization of poly(**4**) and poly(**5**). Figures S1–S13: ^1^H NMR spectra of monomers and polymers. Figures S14–S19: IR spectra of monomers and polymers. Figure S20: UV spectra of monomers. Figure S21: Measurement of a maximum flexural stress (σ/pa). Figure S22: Relationship between α and *P*_O2_ through the membranes of poly(**2**)–poly(**4**). Figure S23: The possible separation mechanism of O_2_/N_2_ through the membranes. Figure S24: XRD of poly(**3**) and poly(**4**) in membrane state. Figure S25: The SEM images of (a) poly(**4**) and (b) poly(**3**) membranes.

## Appendix A

1. We have previously reported these cis-cisoid and cis-transoid conformation can be determined by UV-vis spectroscopy because the conjugation length of the former is shorter than that of the latter [13,14,18,19]. For the extensive consideration, see Ref. [19].
2. If these polymers made hydrogen bonds *inter*molecularly, it also affected their solubility largely. However, we have already reported that these polymers from the monomers having two hydroxy groups (such as **1**, **2** and **4**) had *intra*molecular hydrogen bonds because the long alkyl groups could prevent from forming *inter*molecular hydrogen bonds. Therefore, the effects of the *inter*molecular hydrogen bonds on the solubility is thought to be not large.
3. Since the synthesis of these monomers needed multi-step synthesis, the total yields were not high (5.5–7.7%).
