# Supplementary material for: Synthesis of Cis-Cisoid or Cis-Transoid Poly(Phenyl-Acetylene)s Having One or Two Carbamate Groups as Oxygen Permeation Membrane Materials
Source: Membranes (Basel). 2020 Aug 25;10(9):199. doi: 10.3390/membranes10090199 (PMC7557842; doi:10.3390/membranes10090199)
Supplement: Supplementary file 1 [file membranes-10-00199-s001.pdf]

Supporting Information (SI)

Synthesis of cis-cisoid or cis-transoid poly(phenyl-acetylene)s having one or two carbamate groups as oxygen permeation membrane materials

Yu Zang [1,*], Yinghui Lun [2], Masahiro Teraguchi [3], Takashi kaneko [3], Hongge Jia [1], Xunhai Zhang [1], Fengjuan Miao [4] and Toshiki Aoki [1,3,*],

[1] Key laboratory of polymer matrix composites, Heilongjiang province, College of Materials Science and Engineering, Qiqihar University, Wenhua Street 42, Qiqihar, Heilongjiang 161006, China

[2] Department of Materials and Chemical Engineering, Hunan Institute of Technology, Hengyang, Hunan 421002, China

[3] Chemistry and Chemical Engineering, Graduate School of Science and Technology, Niigata University, Ikarashi 2-8050, Nishi-ku, Niigata 950-2181, Japan

[4] College of Communications and Electronics Engineering, Qiqihar University, Wenhua Street 42, Qiqihar, Heilongjiang 161006, China

* Correspondence: zangyu.25@163.com; toshaoki@eng.niigata-u.ac.jp

Contents

S1. Synthesis of compounds 6-9

S1.1. 4-Bromo-2, 6-bis(hydroxymethyl)-1-phenol (**6**)

S1. 2. 2, 6-Bis (acetoxymethyl)-4-bromo-1-phenyl acetate (**7**)

S1. 3. 2, 6-Bis (acetoxymethyl)-4-(trimethylsilylethynyl)-1-phenyl acetate (**8**)

S1.4. 2, 6-Bis(hydroxymethyl)-4-ethynylphenol (**9**)

S2. Supplemental scheme and figures for the text

S3. The crystallinity and morphology of poly(3) and poly(4) membranes

S3.1 The crystallinity of poly(**3**) and poly(**4**) membranes

S3.2 The morphology of poly(**3**) and poly(**4**) membranes

S4. Unsuitability of the monomers for helix-sense-selective polymerization

S1. Synthesis of compounds 6-9

S1.1. 4-Bromo-2, 6-bis(hydroxymethyl)-1-phenol (**6**)

A formaldehyde aqueous solution (37.0 wt%, 300 mL, 4.00 mol) was added dropwise to a solution of 4-bromophenol (51.1 g, 0.295 mol) and potassium hydroxide (18.5 g, 0.393 mol) in 2-propanol (100 mL), the solution was heated and stirred at 40 ℃ for 89 h. The resulting solution was cooled to room temperature and poured into 0.1 mol/L hydrochloric acid (1000 mL) with stirring. The mixture was allowed to stand for about 6 h to give a red viscous solid as a precipitate. After removing the red solid by decantation, the supernatant solution was allowed to stand for 2 days to precipitate a white solid. The solid was filtered, washed with chloroform, and dried to give a white solid. Yield: 63.4% (43.6 g). ^{1}H NMR (400 MHz, DMSO-d_6, δ): 8.78 (s, 1H, PhO*H*), 7.29 (s, 2H, Ph*H*), 5.33 (t, 2H, $(CH_2OH)_2$), 4.51 (d, 4H, $Ph(CH_2OH)_2$).

S1. 2. 2, 6-Bis (acetoxymethyl)-4-bromo-1-phenyl acetate (**7**)

To a pyridine solution (50.0 mL) of **6** (9.50 g, 40.8 mmol), acetic anhydride (257 mmol, 24.3 mL) was added dropwise at 0 ℃. The solution was stirred for 1.5 h at room temperature and then ethyl acetate (100 mL) was added to the mixture. The mixture was washed with saturated aqueous solution of $CuSO_4{\cdot}5H_2O$ to remove pyridine. The organic layer was dried over anhydrous $MgSO_4$. After concentration, the crude product was purified by silica-gel column chromatography to give a white solid. Yield: 87.8% (12.8 g). R_f = 0.30 (ethyl acetate/hexane = 1/2). ^{1}H NMR (400 MHz, DMSO-d_6, δ): 7.68 (s, 2H, Ph*H*), 4.97 (s, 4H, $Ph(CH_2O)_2$, 2.31 (s, 3H, $PhOCOCH_3$), 2.03 (s, 6H, $Ph(CH_2OCOCH_3)_2$).

S1. 3. 2, 6-Bis (acetoxymethyl)-4-(trimethylsilylethynyl)-1-phenyl acetate (**8**)

A mixture of **7** (12.8 g, 35.7 mmol), triphenylphosphine (672 mg, 2.64 mmol), copper (I) iodide (832 mg, 4.44 mmol), bis(triphenylphosphine)palladium(II) dichloride (521 mg, 0.730 mmol) and trimethylsilylacetylene (7.10 mL, 50.2 mmol) in triethylamine (143 mL) was refluxed for 24 h. And then the mixture was filtered, the solvent was removed by evaporation. The crude product was purified by silica-gel column chromatography to give **8** as a brown liquid. Yield: 85.0% (11.4 g). R_f = 0.34 (ethyl acetate/hexane = 1/3). ^{1}H NMR (400 MHz, $CDCl_3$, TMS, δ): 7.50 (s, 2H, Ph*H*), 4.98 (s, 4H, $Ph(CH_2OAc)_2$), 2.32 (s, 3H, $PhOCOCH_3$), 2.06 (s, 6H, $Ph(CH_2OCOCH_3)_2$), 0.22 (s, 9H, $Si(CH_3)_3$).

S1.4. 2, 6-Bis(hydroxymethyl)-4-ethynylphenol (**9**)

To a mixture of lithium aluminum hydride (1.15 g, 30.2 mmol) and tetrahydrofuran (50.0 mL), a tetrahydrofuran solution (10.0 mL) of **8** (5.70 g, 15.1 mmol) was added dropwise at 0 ℃. The mixture was stirred for 2h at room temperature, and then deionized water (55.0 mL) was added dropwise to the reaction mixture at 0 ℃. The mixture was stirred for an additional 12 h at room temperature. The reaction mixture was treated with 2N HCl aqueous to precipitate aluminum salts. After filtration of the solid, tetrahydrofuran was removed by evaporation from the filtrate. The product was dissolved in ethyl acetate and the solution was washed with water. The organic layer was dried over anhydrous $MgSO_4$ and then was concentrated by evaporation. The crude product was purified by silica-gel column chromatography to give **9** as a white solid. 76.6% (2.13 g). R_f = 0.27 (ethyl acetate/hexane = 3/2). ^{1}H-NMR(400MHz, DMSO-d_6, δ): 8.98(s, 1H, PhO*H*), 7.27 (s, 2H, Ph*H*), 5.29 (t, 2H, $Ph(CH_2OH)_2$), 4.52 (d, 4H, $Ph(CH_2OH)_2$), 3.91 (s, 1H, *H*C≡C).

S2. Supplemental scheme and figures for the text

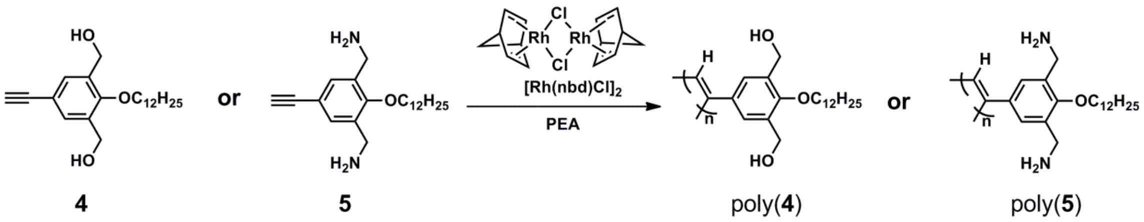

Scheme S1. Synthetic route to poly(**4**) and poly(**5**).

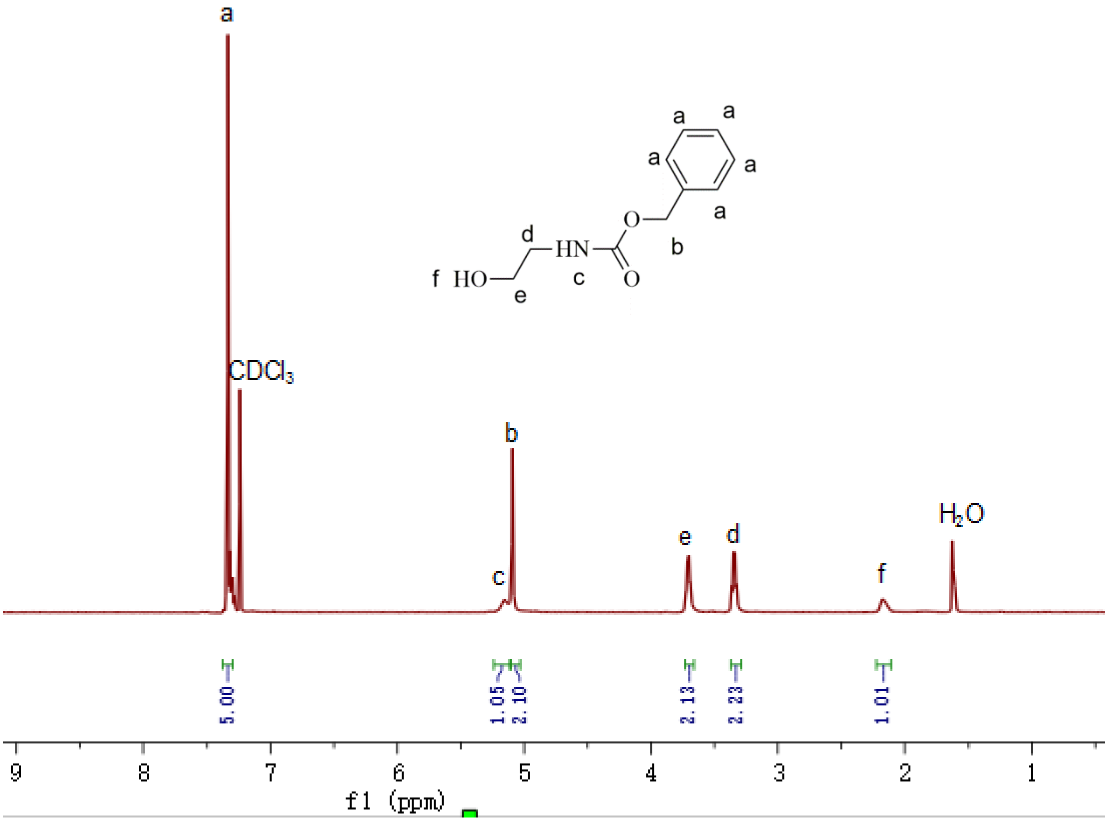

Figure S1 ^{1}H NMR spectrum of N-benzyloxycarbonyl-2-aminoethanol (**10**, *m*=2) (Scheme 1)

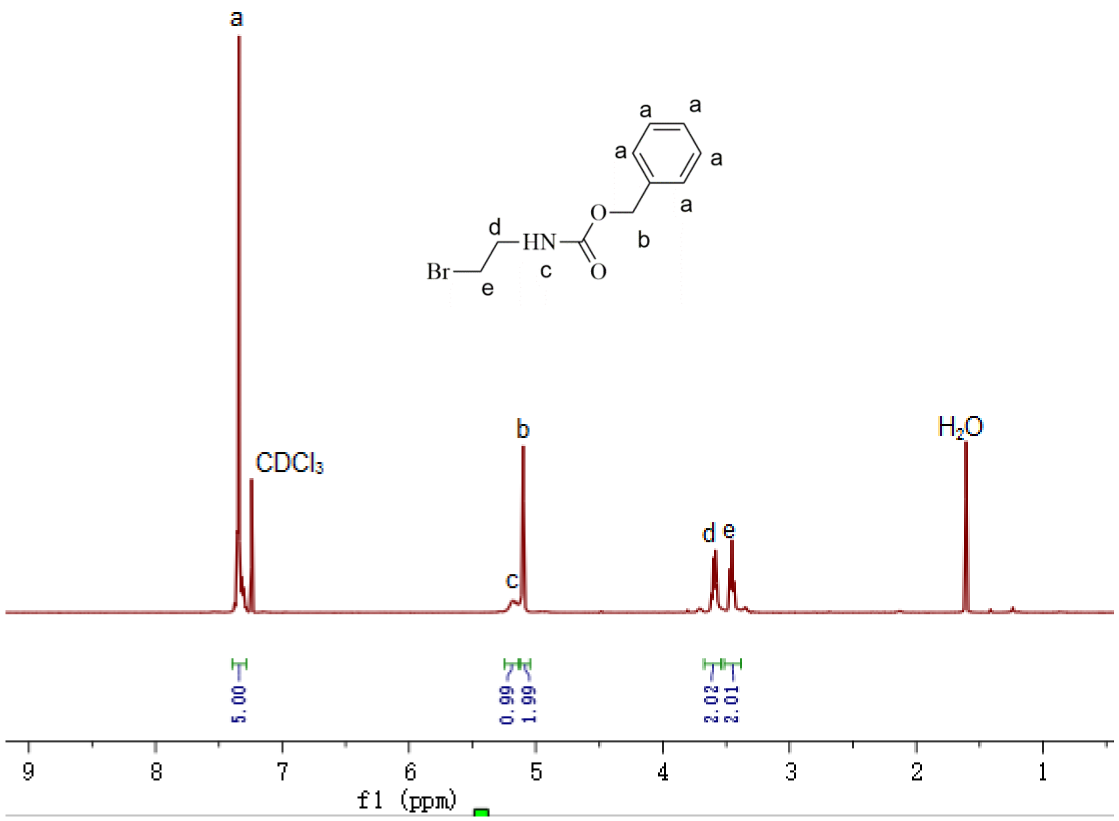

Figure S2. ^{1}H NMR spectrum of N-benzyloxycarbonyl-2-bromoethylamine (**10**, *m*=6) (Scheme 1)

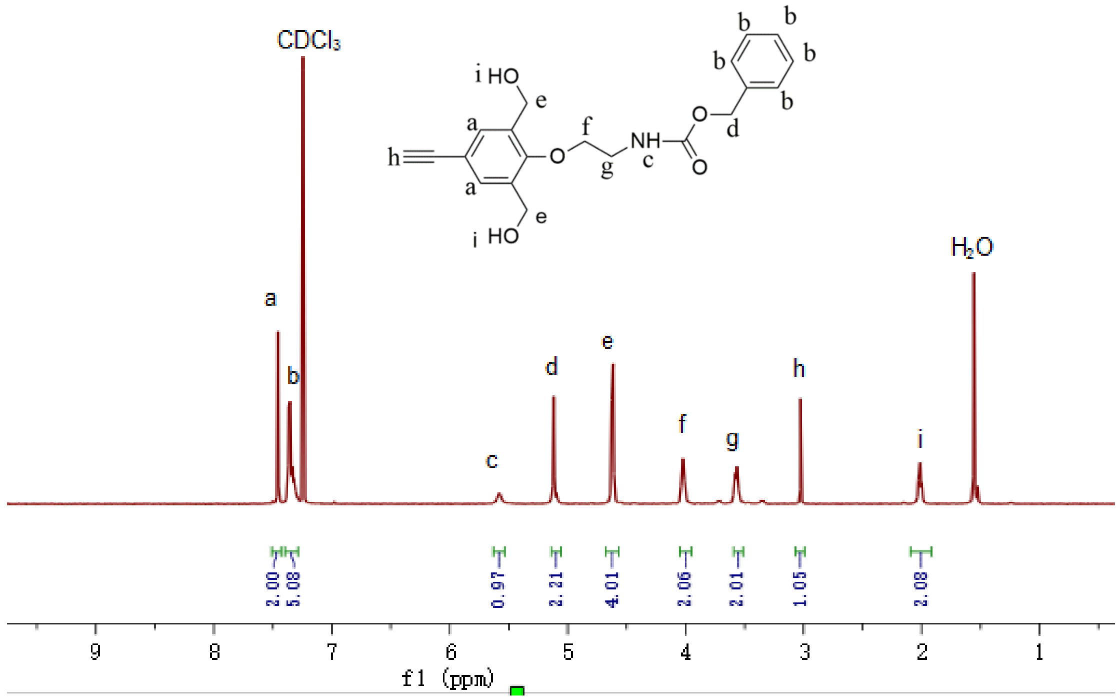

Figure S3. ^{1}H NMR spectrum of **1** (Scheme 1)

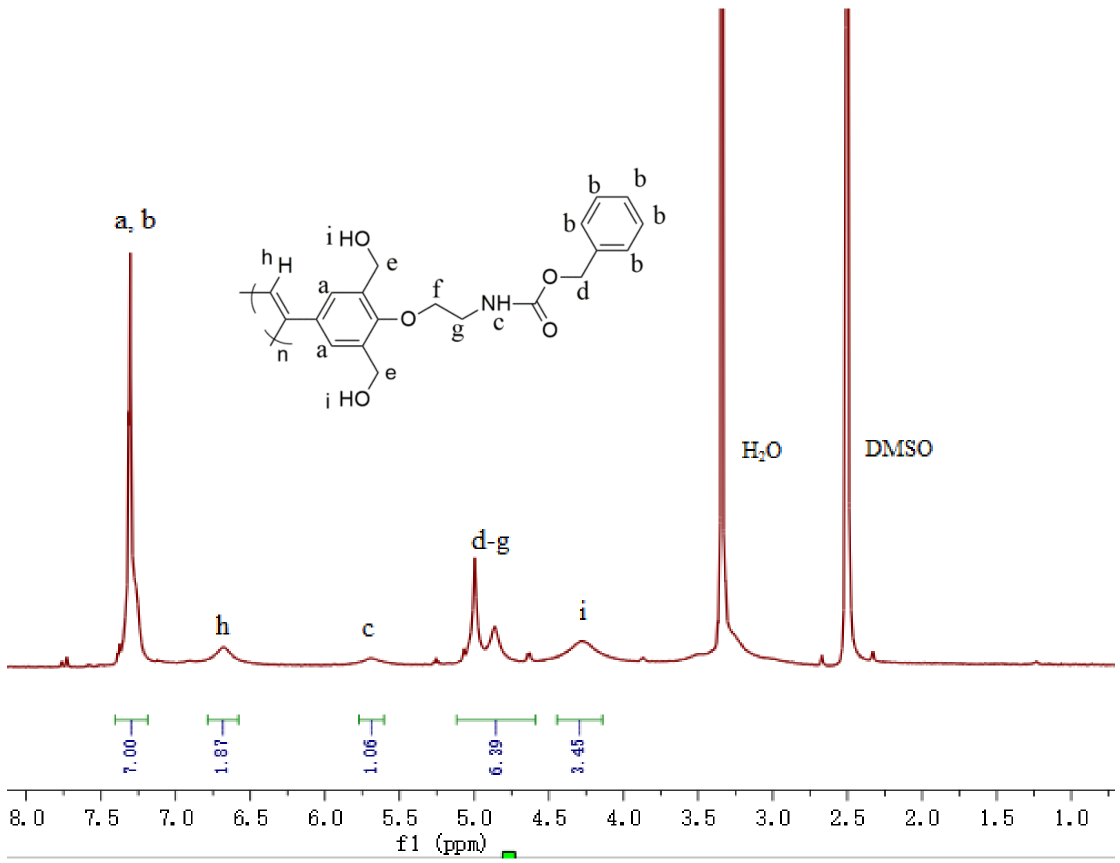

Figure S4. ^{1}H NMR spectrum of poly(**1**)(Scheme 1)

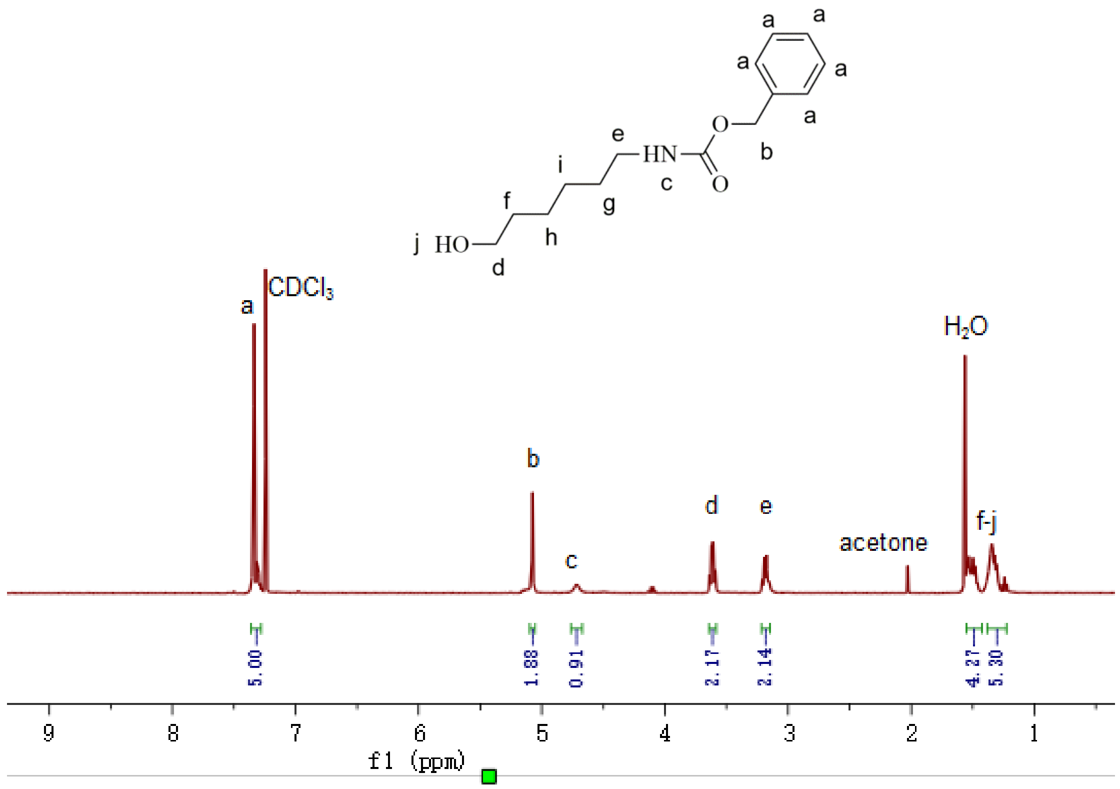

Figure S5. ^{1}H NMR spectrum of N-(benzyloxycarbonyl)-6-amino-1-hexanol (**11**, *m*=6) (Scheme 1)

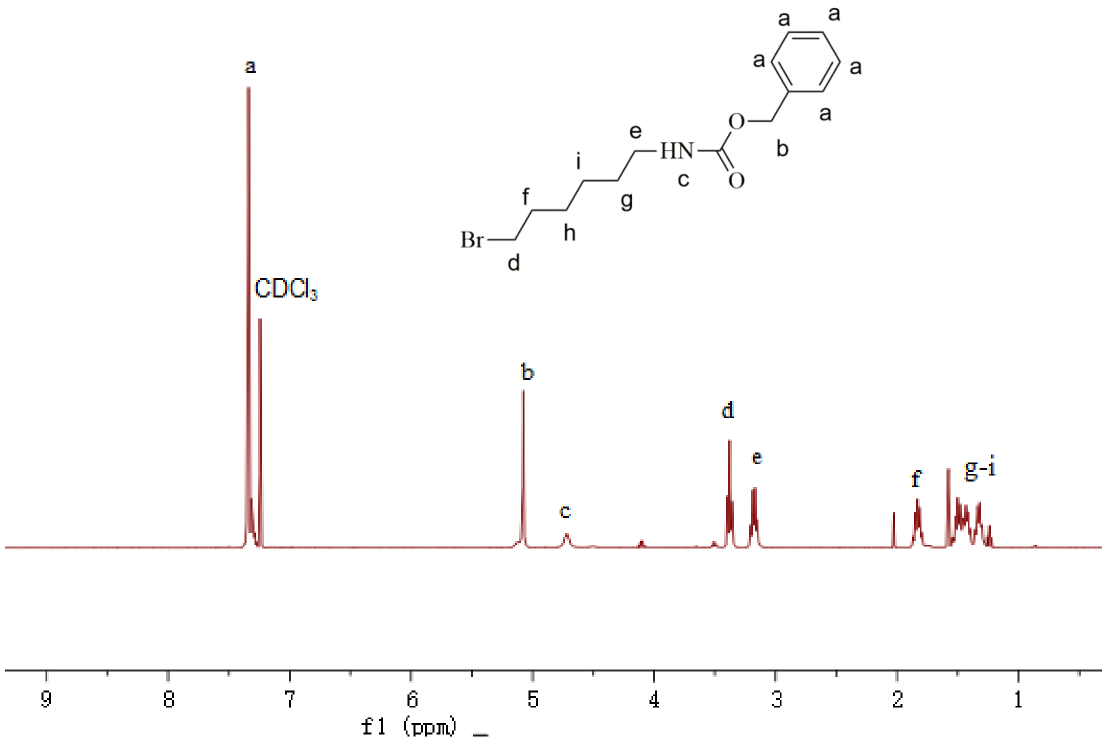

Figure S6. ^{1}H NMR spectrum of N-(benzyloxycarbonyl)-6-bromohexylamine (**11**, *m*=6) (Scheme 1)

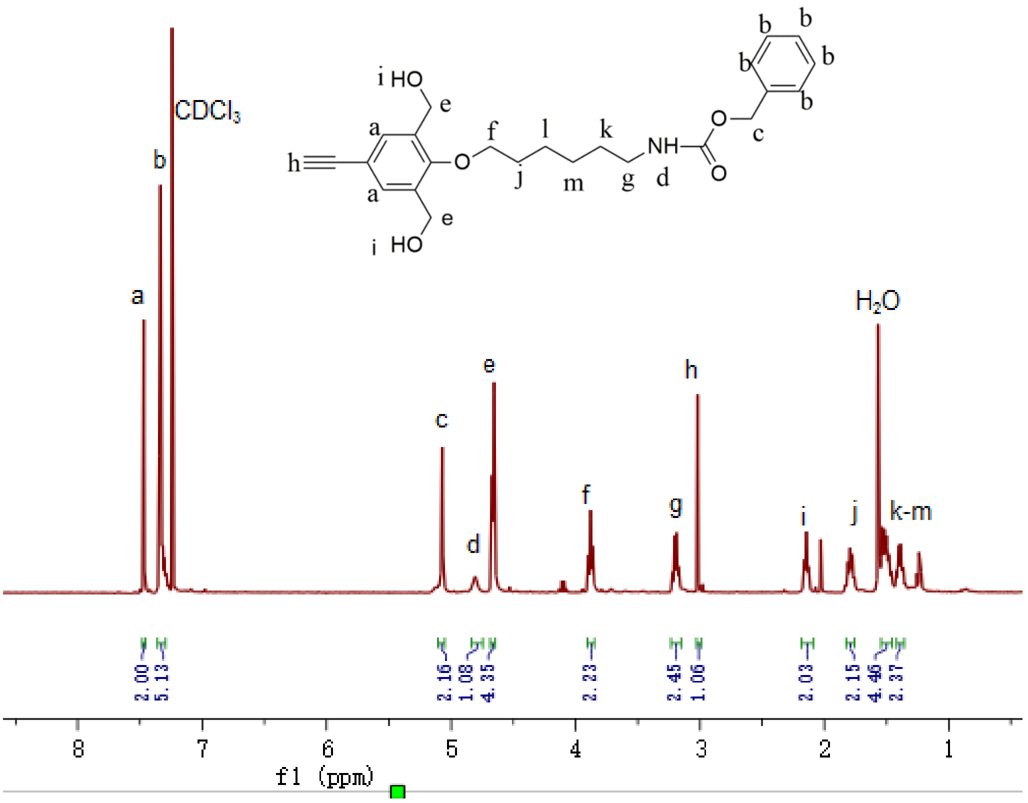

Figure S7. ^{1}H NMR spectrum of **2** (Scheme 1)

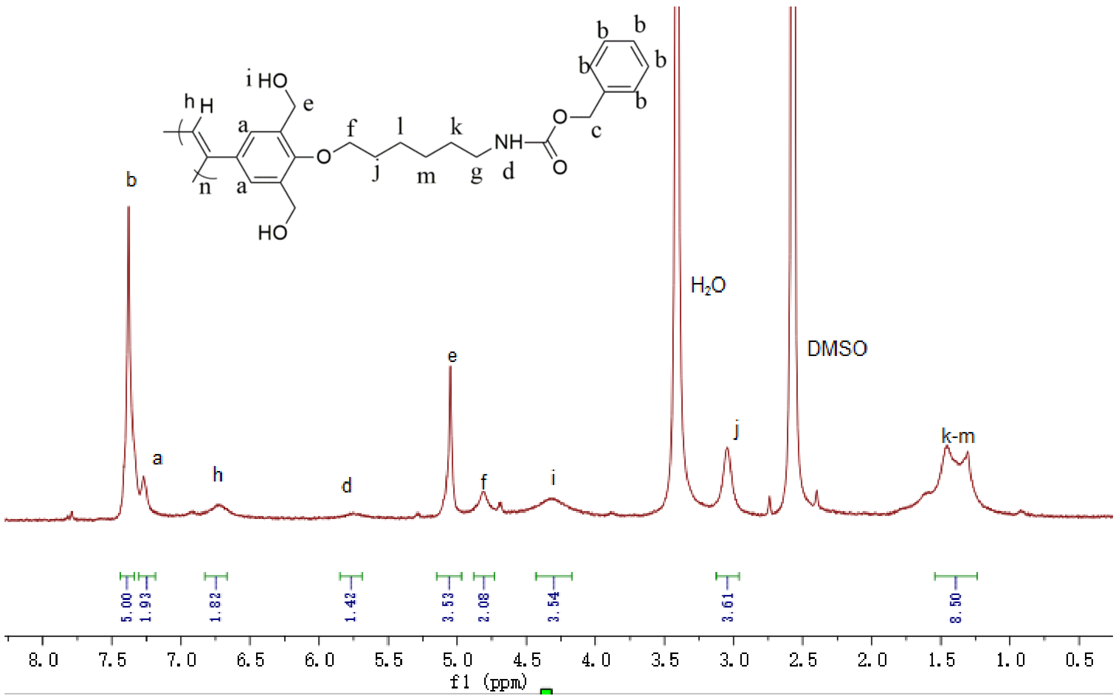

Figure S8. ^{1}H NMR spectrum of poly(**2**) (Scheme 1)

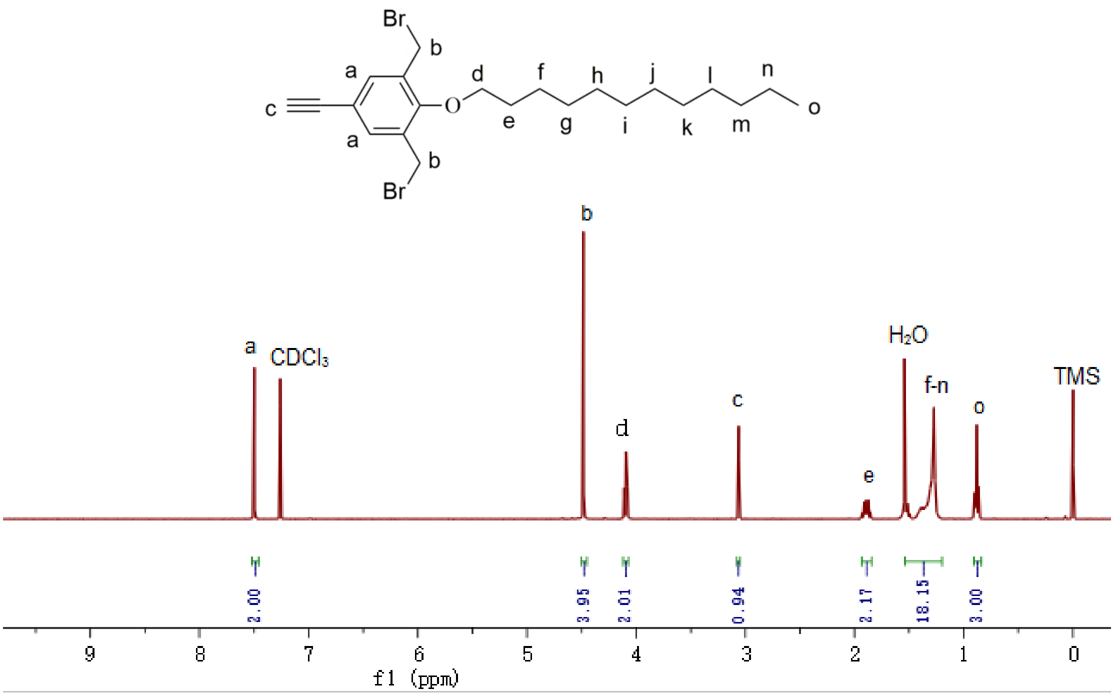

Figure S9. ^{1}H NMR spectrum of 4-dodecyloxy-3,5-bis(bromomethyl)phenylacetylene(**12**) (Scheme 2)

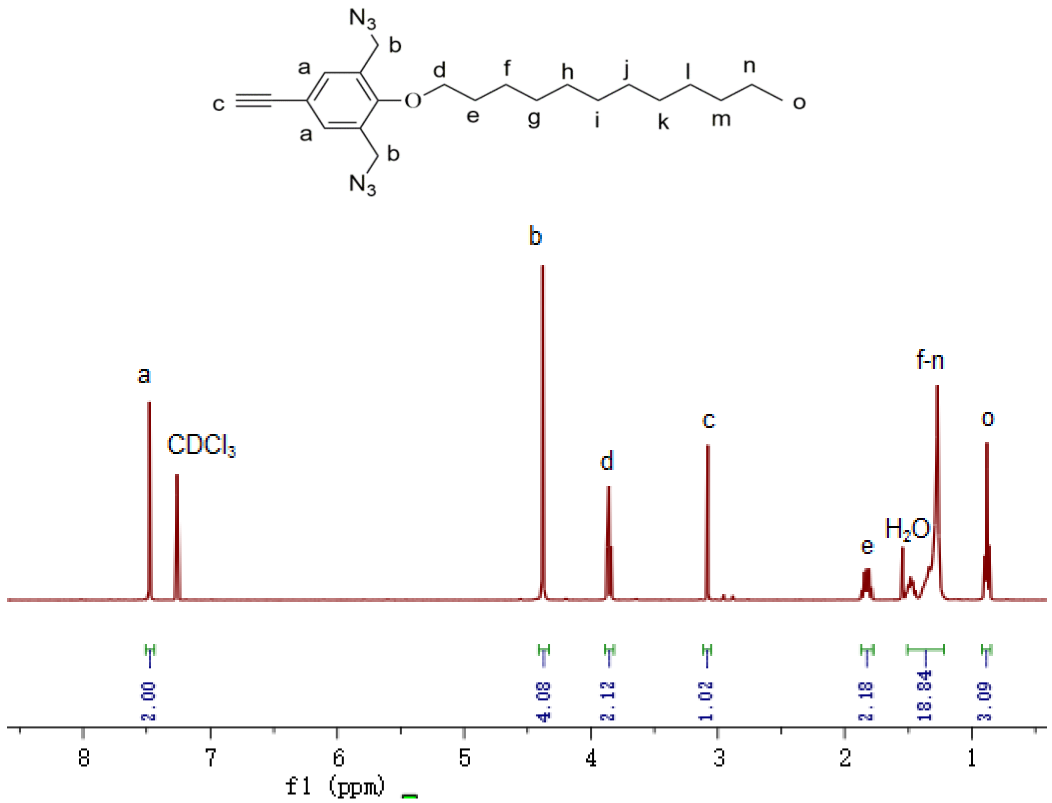

Figure S10. ^{1}H NMR spectrum of 4-dodecyloxy-3,5-bis(nitrinemethyl)phenylacetylene(**13**) (Scheme 2)

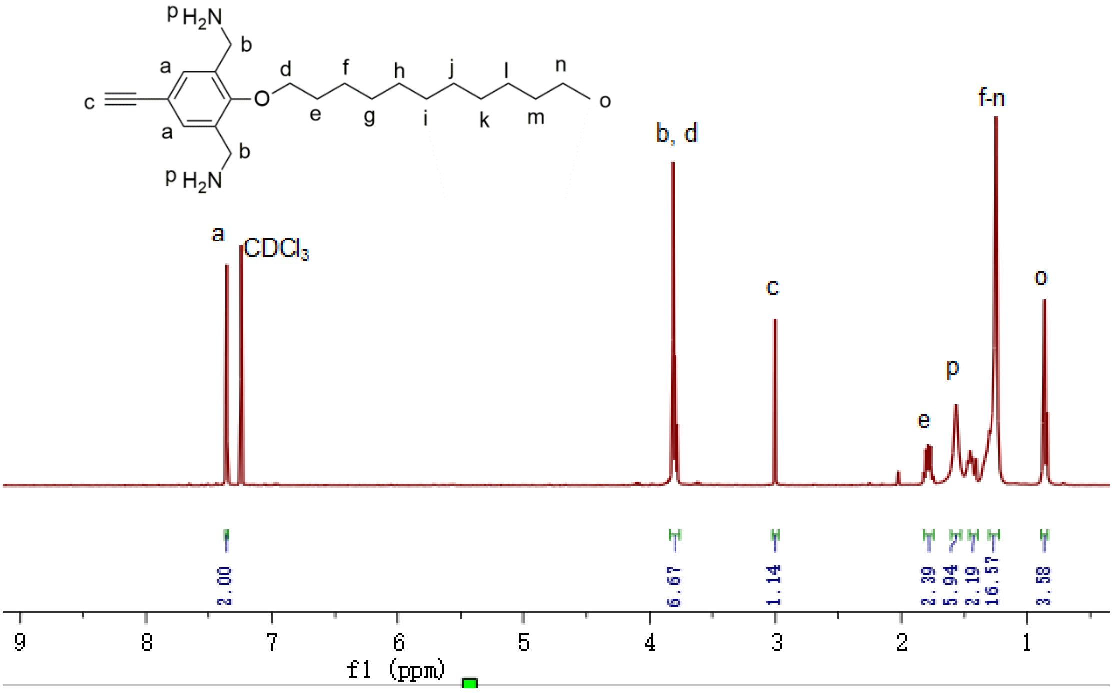

Figure S11. ^{1}H NMR spectrum of **5** (Scheme 2)

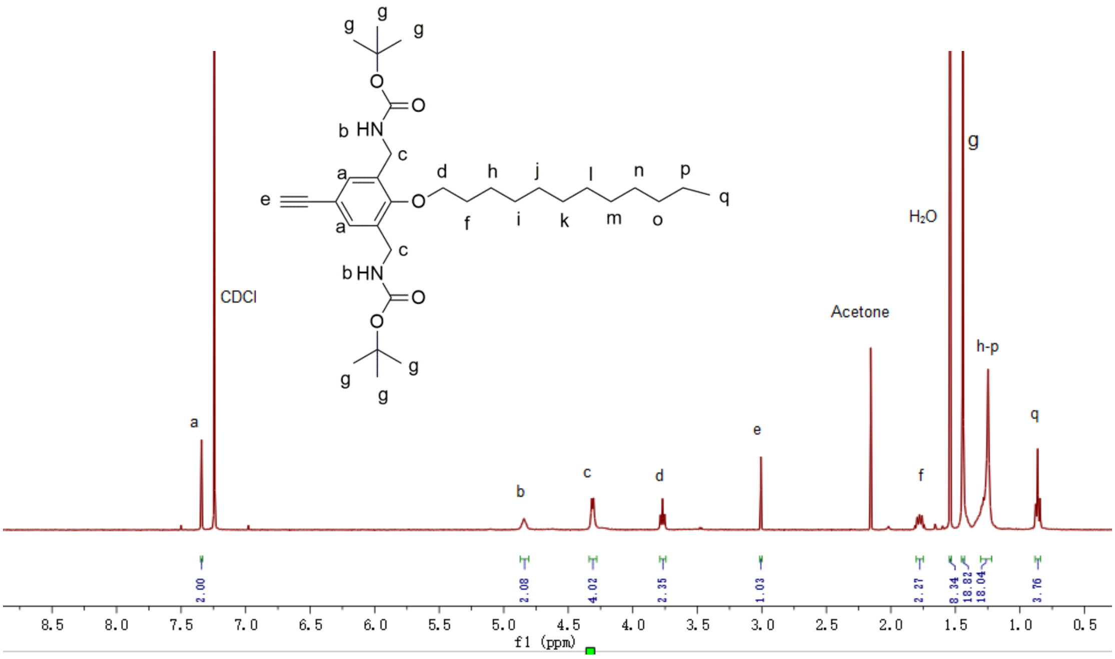

Figure S12. ^{1}H NMR spectra of **3** (Scheme 2)

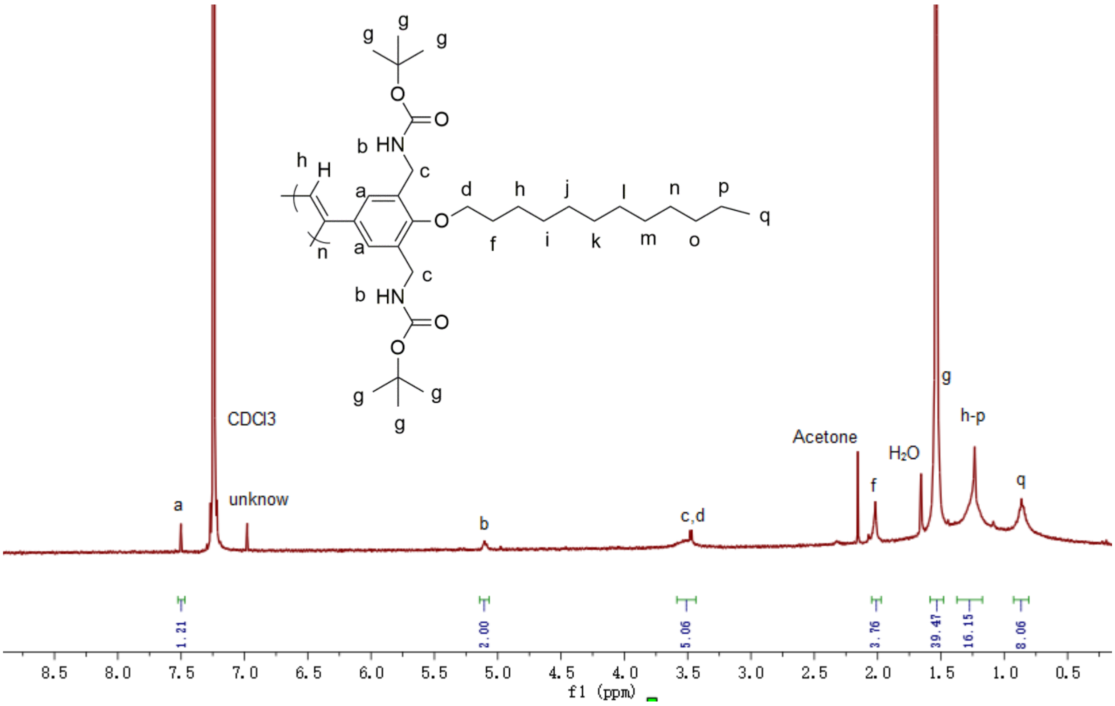

Figure S13. ^{1}H NMR spectra of poly (**3**) (Scheme 2)

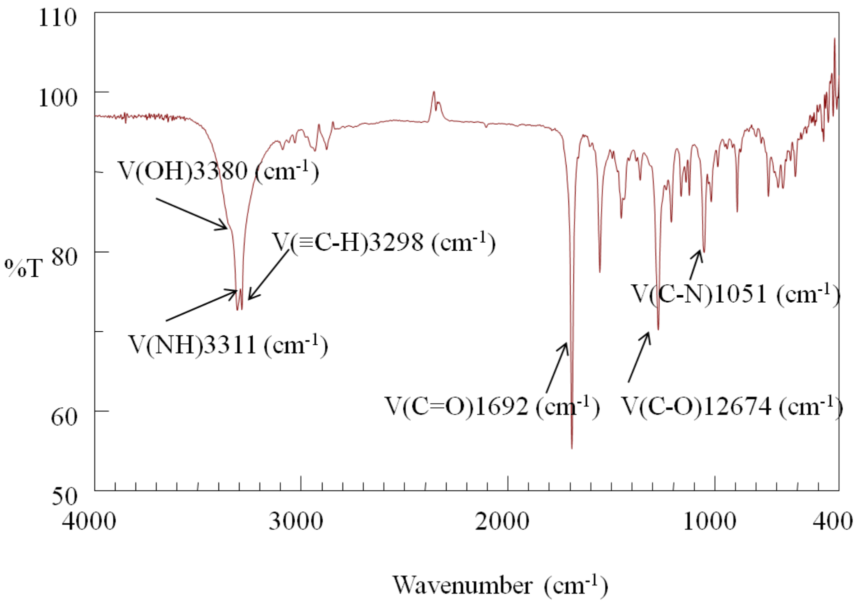

Figure S14. IR spectra of **1** in solid state (KBr)

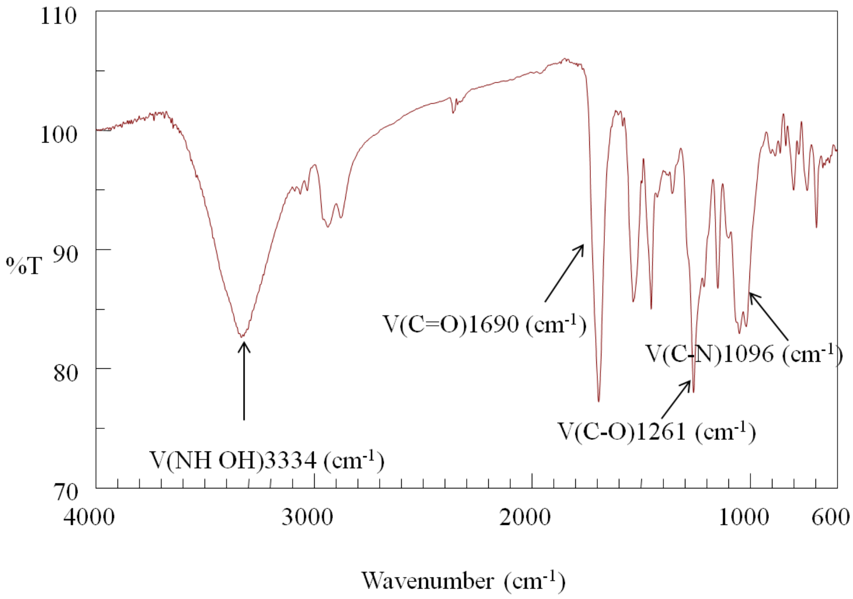

Figure S15. IR spectra of poly(**1**) in solid state (KBr)

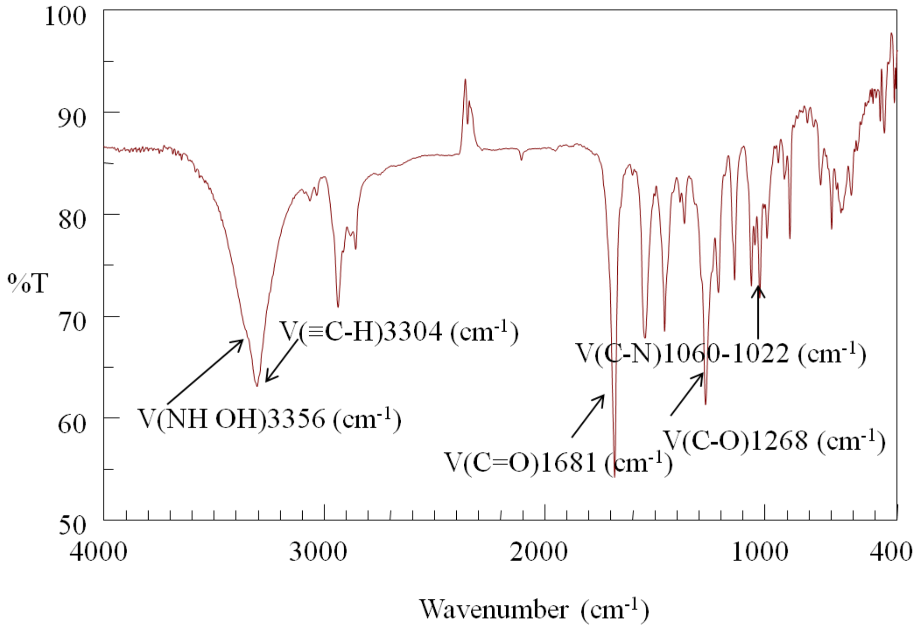

Figure S16. IR spectra of **2** in solid state (KBr)

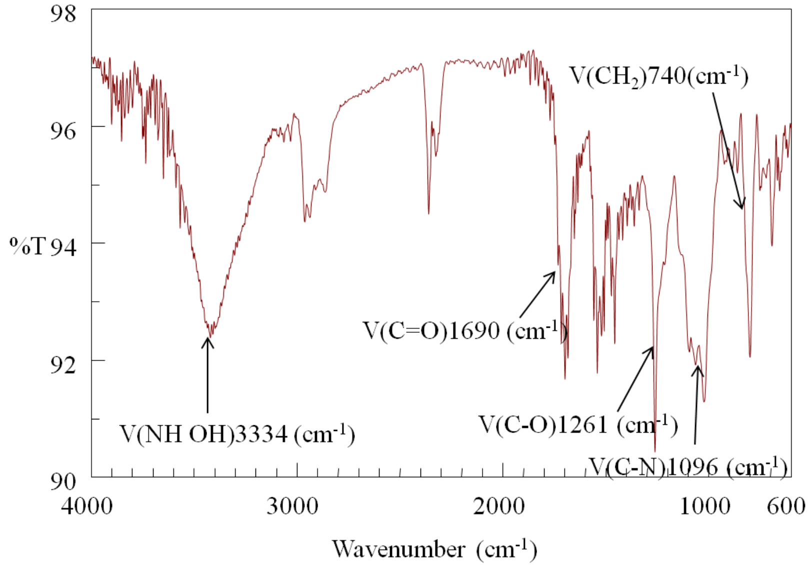

Figure S17. IR spectra of poly(**2**) in solid state (KBr)

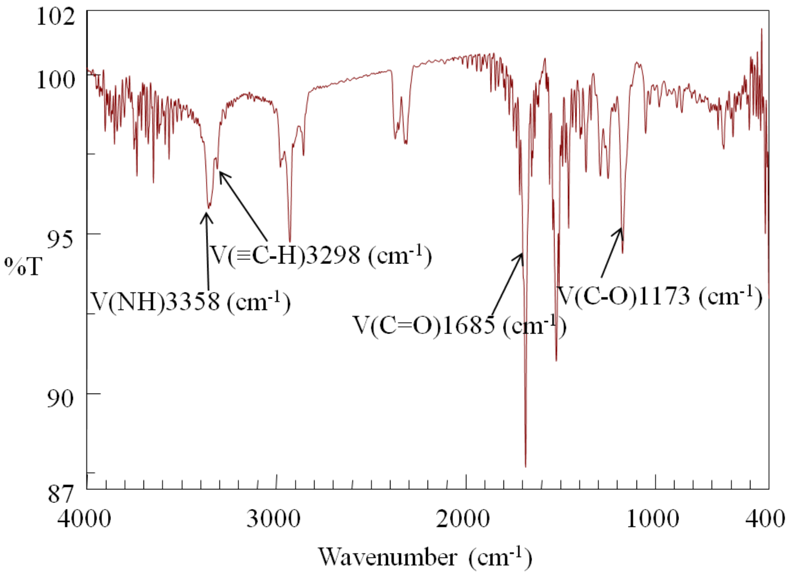

Figure S18. IR spectra of **3** in solid state (KBr)

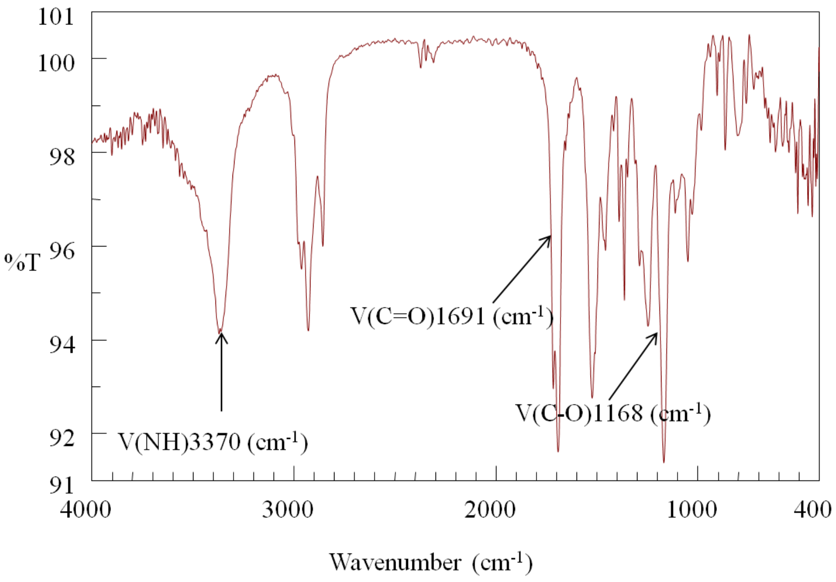

Figure S19. IR spectra of poly(**3)** in solid state (KBr)

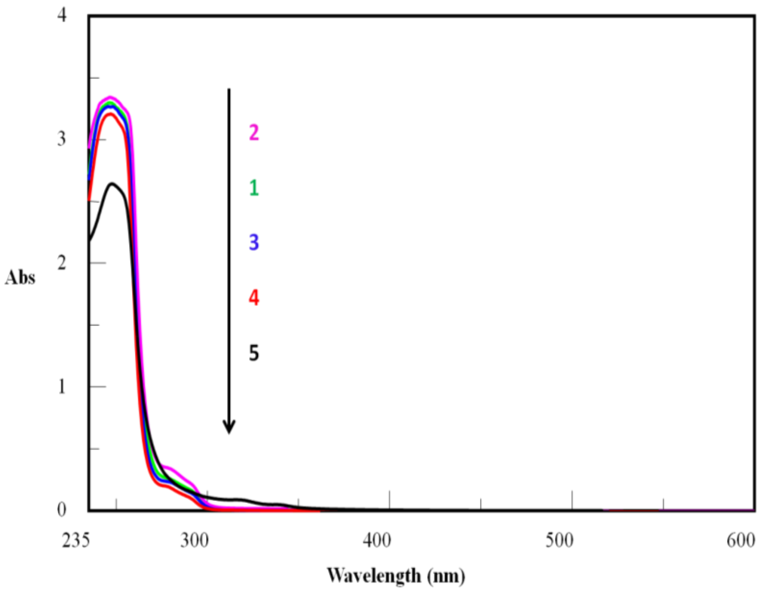

Figure S20. UV spectra of monomers **1-5** in $CHCl_3$ (c=2.00mmol/L)

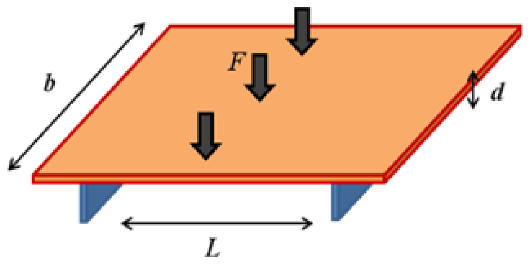

$$\sigma_{(Pa)} = \frac{3F_{(N)}L_{(m)}}{2b_{(m)}d^2_{(m^2)}}$$

Figure S21. Measurement of a maximum flexural stress (σ/pa).

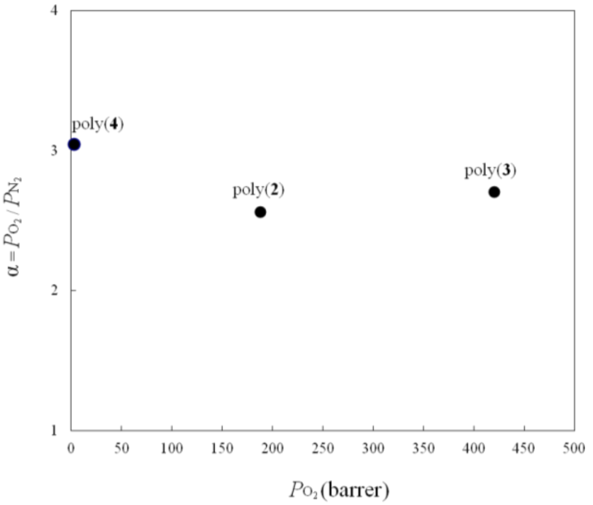

Figure S22. Relationship between α and PO_2 through the membranes of poly(**2**)-poly(**4**).

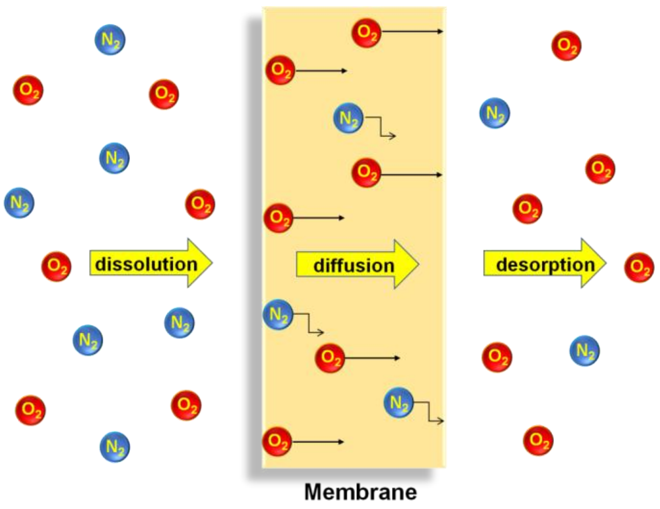

Figure 23. The possible separation mechanism of O_2/N_2 through the membranes.

S3. The crystallinity and morphology of poly(3) and poly(4) membranes

S3.1 The crystallinity of poly(**3**) and poly(**4**) membranes

The poly(**3**) membrane which took loose cis-transoidal racemic helical main chains shows much lower crystallinity than that of poly(**4**) whose main chain took regular cis-cisoidal helical conformation. The columnar diameter D and lattice spacing of poly(**3**) membrane are smaller than that of poly(**4**) due to the loose cis-transoidal conformation.

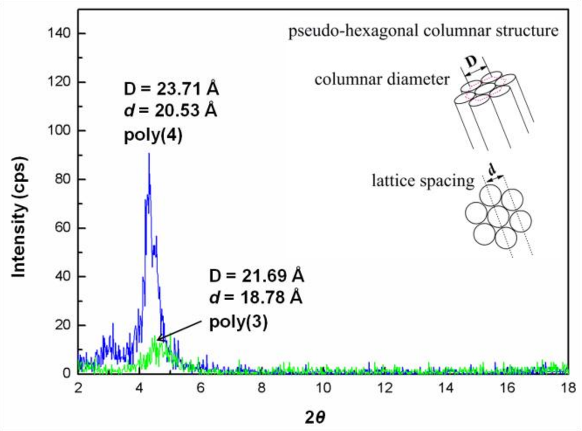

Figure S24. XRD of poly(**3**) and poly(**4**) in membrane state.

S3.2 The morphology of poly(**3**) and poly(**4**) membranes

The surface morphology of the poly(**3**) and poly(**4**) membranes were measured by Scanning Electron Microscope (SEM) which were recorded on a HITACHI S-4300 electron microscope. As shown in Figure S25, the surface of poly(**3**) membrane is more smooth than that of poly(**4**). The higher solubility of poly(**3**) gave a good dense membranes without defect.

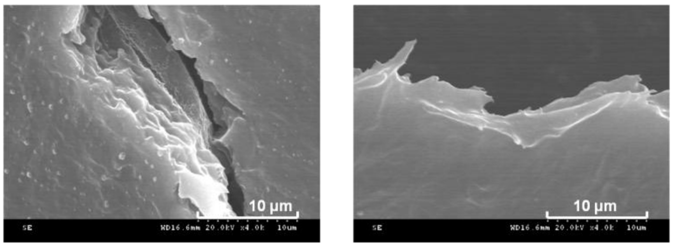

（a） （b）

Figure S25. The SEM images of (a) poly(**4**) and (b) poly(**3**) membranes.

S4. Unsuitability of the monomers for helix-sense-selective polymerization

As we described in the introduction part, we reported before that monomer **4** having two hydroxymethyl groups was suitable for helix-sense-selective polymerization because the resulting polymer could tight cis-cis helicity stabilized by intramolecular hydrogen bonds between OH groups [13]. However, the three new monomers **1**-**3** were not suitable for helix-sense-selective polymerization because no Cotton effect was observed in the resulting three new polymers (poly(**1**)-poly(**3**)). Although monomers **1** and **2** containing two hydroxy groups similar to monomer **4**, by the introduction of the carbamate group, the polarity of the polymers became higher and therefore they could soluble only in polar solvents where hydrogen bonds were not stable. As a result the intramolecular hydrogen bonds between OH groups in the polymer were disrupted and became weaker (Figure 3). Therefore, it could not maintain one-handed helicity, although they had tight cis-cisoidal conformations. In order to discuss hydrogen bonds in the new polymers, the IR spectra of poly(**1**) and poly(**2**) together with poly(**4**) which has two hydroxy groups in their monomer unit were measured in $CHCl_3$ (2.00 mmol/L)(Figure 3). The stretching vibration bands of O-H were observed around 3336 and 3337cm^{-1} for poly(**1**) and poly(**2**), respectively. Since the stretching vibration band of O-H for poly(**4**) having no carbamate groups appeared at 3309 cm^{-1}, the introduction of the carbamate groups weekend the hydrogen bonds between the hydroxy groups. As results, poly(**1**) and poly(**2**) prepared by using chiral cocatalysts had no CD peaks different from poy(**3**) because their main chain stability was a littler lower.

In the case of poly(**3**), the two bulky t-butyl groups may prevent the forming of intramolecular hydrogen bonds between NH and CO groups (Figure 4). Therefore, no Cotton effect could be found in poly(**3**). As another possible reason for the unsuitability for helix-sense-selective polymerization of the carbamate-containing monomers, the interaction between the carbamate group and rhodium catalyst could have affected the formation of stable one-handed helical main chain or the function of the polymerization catalyst.
